# A hybrid Forecast Cost Benefit Classification of diabetes mellitus prevalence based on epidemiological study on Real-life patient’s data

**DOI:** 10.1038/s41598-019-46631-9

**Published:** 2019-07-12

**Authors:** Muhammad Noman Sohail, Ren Jiadong, Musa Muhammad Uba, Muhammad Irshad, Wasim Iqbal, Jehangir Arshad, Antony Verghese John

**Affiliations:** 1 Department of Information Sciences and Technology, Yanshan University, Hebei, China; 2 Department of Economics and Management, Yanshan University, Hebei, China; 3 Department of Computer Sciences and Technology, Comsat University, Islamabad, Pakistan; 4 Department of Hotel Management, American Hotel and Lodging Association, New York, United States of America

**Keywords:** Lifestyle modification, Health care economics, Patient education, Experimental models of disease

## Abstract

The increasing ratio of diabetes is found risky across the planet. Therefore, the diagnosis is important in population with extreme risk of diabetes. In this study, a decision-making classifier (J48) is applied over a data-mining platform (Weka) to measure accuracy and linear regression on classification results to forecast cost/benefit ratio in diabetes mellitus patients along with prevalence. In total 108 invasive and non-invasive medical features are considered from 251 patients for assessment, and the real-time data are gathered from Pakistan over a time span of June 2017 to April 2018. The results indicate that J48 classifiers achieved the best accuracy of (99.28%), whereas, error rate (0.08%), Kappa stats, PRC, and MCC are (0.98%), precision, recall, and F-matrix are (0.99%). In addition, true positive rate is (0.99%) and false positive is (0.08%). The regression forecast decision indicates blood pressure and glucose level are key features for diabetes. The cost/benefit matrix indicates two predictions for positive test with accuracy (66.68%) and (30.60%), and key attributes with total Gain (118.13%). The study confirmed the proposed prediction is practical for screening of diabetes mellitus patients at the initial stage without invasive medical tests and found effectual in the early diagnosis of diabetes.

## Introduction

The data mining is a modern technique of digging into data to locate patterns ideally. It is an astounded spectator of incredible progress in modern health care^1^. The undiscovered electronic health records (EHRs) are being used to manipulate the unknown patterns located in the health datasets and healthcare industries, which are enthusiastically supporting the copious amount of data. This data can be manipulate for prognostication and scrutinizing of diabetes mellitus ratio in epidemiological states around the globe^2^. The data mining techniques contain the association rule mining for finding the outlines of modern classifications and cluster. From the last several years, data processing methods are being utilized in various domains by choosing data from different machine learning repositories but it came extremely late in diabetes section^3^. The data mining platform is supported by divine mysteries including machine learning 60%^4^, statistics 30%^5^, artificial intelligence 5%^6^ and probabilities 5%^7^. Study indicates that the data mining techniques have been used to diagnose diabetes mellitus in developing countries, i.e. Ashrafuzaman *et al*. mentioned that diabetes is properly considered as most rapidly spreading and inaudibly life-taking disease that causes the most valued snags in the modern world of health^8^. According to the initial predictions, diabetes preserves serious health complications as mentioned in the book “Heavens on Earth”^9^. However, the accurate predictions of diabetes remain a perplexing task for the medical whizzes due to its clandestine patterns.

According to (NIH), the number of patients suffering from diabetes is increasing exponentially that leads with some causes but the most significant factors include age, diet plans, glucose levels, sugar intakes, thirst, urination, vision symptoms, skin issues, and others with body mass indexes^10,11^. The mostly discussed level of diabetes mellitus found are Type-2 however, Type-1 diabetes and Gestational are at its peak in the developing countries, which are affecting the other severe health complications i.e. kidney, heart attack, strokes, and cancer. In addition, 20% of patients are treated with insulin injections to control their glucose and blood pressure levels^12^. However, if it is neglected, it can cause slowly spreading germs to the profound effect the optimal health. Williams wrote in his book ‘Williams textbook of Endocrinology’^13^ that around 385 million people were affected with diabetes in 2013 and this ratio can get higher if it remains untreated and can indeed cause to death. Around 425 million people are suffering from diabetes around the globe mentioned by the survey report of the International Diabetes Federation (IDF) in 2015^14^. The report also indicates that almost 382 million people are affected by diabetes only in developing countries out of which 4.9% are from Asia. According to World Health Organization (WHO)^15^, 3.31 million deaths occurred in Asia region due to diabetes; out of which 79% population is under age of 55 that is the maximum number in any region of the world. The ratio of diabetes mellitus patients in rural and urban areas of Pakistan varies from 0.56% to 12% and it has been estimated more than double of this ratio over the past two decades^16^.

Numerous authors worked on different techniques for the classification of diabetes mellitus like Raj Bala *et al*. worked for missing values and unclear facts and analyzed the figurative rule mining worktable for spawning initial rule sets and control the projects to discover the rule sets^17^. Rojas *et al*. investigate on the theory of linear genetic classification^18^. Saini *et al*. worked on Unsupervised filtering by SVM principle^19^. In addition, he introduced a structure centered by heterogeneous and grid distribution. Noman *et al*. talked about the semi supervised clustering approach by utilizing K-means and SOM algorithms^20^. Andrew *et al*. worked with outlier prediction method to accurately diagnose cardiovascular diseases^21^. By report of Sohail *et al*.^22^ Xue applied a Bayesian algorithm for spotting of confusion Coronary Cardiopathy. In addition, Xing predicted the survival of CHID patients by biomedical images. Moreover, he mentioned that Shusaku *et al*. worked by Term-Mapping for data processing of multi-scale harmonizing^23^. Muhammad *et al*. deliberated the qualities of choosing Persecution with K-means to categorize the diabetes datasets and presented an approach for clinical screening^24^. Various other data mining techniques were determined by authors in classification and prediction of diabetes mellitus, which are presented in “Table 1” with their accuracy ratios.

**Table 1:** Show the accuracy of Machine-learning algorithms performed for diabetes classification.

Acc %	DMAT	Year	Data	Ref	Author
90	Bayesian NN	2014	Artificial data utilized from UCI repositories	^3^	H. Das
71	PCA with NN	2013		^3^	Jabbar
94	KNN	2016		^3^	Mangathayaru
89	ANN	2015		^3^	A. Kumar
76	Naive Bayes	2015		^3^	A. Lyer
86	C 4.5	2014		^3^	F. Sambo
98	J48	2017		^28^	O. Barrios
94	FCM-WEKA	2017		^3^	Poongothai
75	BLR-Tangra	2014		^3^	S. Bashir^a^

^a^Acc = accuracy, % = percentage, DMAT = data mining algorithms and techniques, Ref = references.

The objective of this study is to develop an accurate prediction assessment model by utilizing intrusive and non-intrusive medical features to identify a high risk of diabetes in the patients. For this purpose, the machine learning classifier J48 decision tree is used on the data-mining platform Weka version 3.9.2 to acquire accuracy in classification assumptions. Subsequently, Linear Regression LR is utilized on classification results to predict and forecast the patients with a high risk of diabetes. The proposed method can be applied to diabetes mellitus patients specifically in the remote areas or villages with low socioeconomic status and extreme epidemiological risk.

## Results

In total, 281 diabetes mellitus patients were evaluated out of 563 for the assessment, including 160 (56.93%) female members and 121 (43.06%) male members. Among 281 records, 11(3.91%) were T2DM (insulin dependent), 256(91.10%) were T1DM (non-insulin dependent), and 14(4.98%) were Gestational. Initially, the real-life dataset was divided into the ratio of 20:80 percent to conduct training and testing. After training the machine, 10-fold cross-validation technique was implemented on an experimental platform of Weka (3.9.2) for classification. The dataset was further divided into 10 samples. Each sample was utilized as validation data from the retention process, while the remaining nine samples served as training data and the process was performed 10 times. This process provided a significant reduction in the error ratio and bias correlation by random sampling.

### Measurements

**Figure 1:**
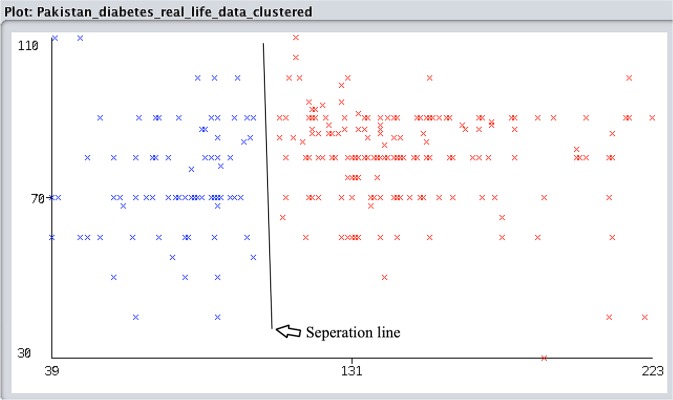
Show the K-means clustering assessment of positive and negative clusters separated by the separation line.

Initially, the K-means clustering algorithm was used for the assessment of positive and negative clusters. K-means provides the specific set of accurate observations, where each observation represents d-dimensional vectors to partition the “n” observations into (*k* ≤ *n*) sets by (1). “Figure 1” presents the classified instance ratio of tested positive and negative clusters with the separation between them. By the assessment of 281 instances, 143 (51%) patients classified as tested positive for diabetes and 138 (49%) classified as tested negative that means the negative instances have fewer symptoms of diabetes and positive has strong.

1 $${\arg }({\min })\sum _{j=1}^{k}\,\frac{1}{2|S|}\sum _{x,y\in S}\Vert x-y\Vert $$

**Figure 2:**
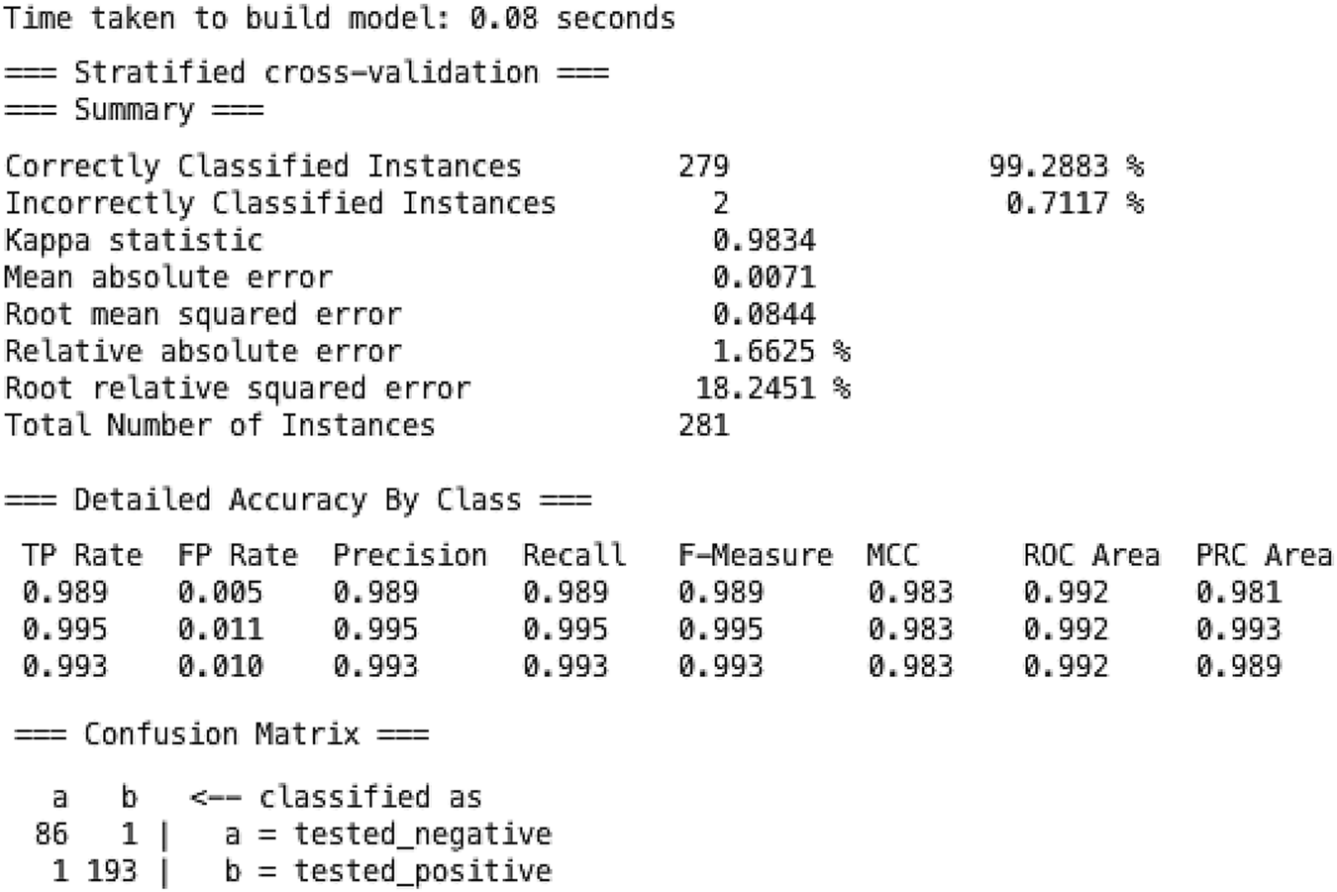
Show the classification assessment of machine learning J48 classifier with the confusion matrix of tested positive and tested negative instances.

In addition, J48 machine learning classification algorithm was utilized on 281 diabetes patients by 10 fold cross-validation technique with parameters as 100 for batch size, 0.25% for the confidence interval, minimum object numbers and decimal number places of 2, 3 numbers of folds with error pruning rate of false, and 1 number of seeds value. After the loop test, the final accuracy reached 99.28% by (2) and the error rate was 0.08%. Weka constructed a J48 evaluation of 0.08 second, a kappa stat result of 0.98%, an average true positive rate of 0.99%, an average false positive rate of 0.08%, an average accuracy, a recall rate, an F matrix, and a ROC area ratio of 0.99%, and The average PRC area ratio, MCC value is 0.98%, as shown in “Fig. 2”.

2 $$Accuracy=(true-ve)+(true+ve)/total$$

Conclusively, “Table 2” divides the final assessment into two parts. Initially, it describes the clustering results achieved by K-means; furthermore, it shows the J48 pruning tree assessment for patients screening. The decision tree indicates that glucose and blood pressure are key features of diabetes screening after the Age attribute. The filtering rules implemented by J48 pruning tree are:

1. If the patient’s glucose level is accurate (>101), diabetes will be predicted to be positive, and in our data set, 191 patients were tested positive because their glucose levels exceeded 101.
2. If the glucose level is accurate (≤101) than 4 cases apply:

i. Check blood pressure, if accurate (≤100), the test patient is negative, in our data set, 85 patients were negative in this particular case.
ii. If blood pressure is usually (>100), the glucose level helps predict, as follows.
iii. If glucose is (≤72), the test is accurately predicted to be negative for diabetes, which is 2 in our data set.
iv. If glucose is (>72) than the specific prediction for the diabetes test is carefully taken as positive, which comes as 3 patients in our data set.

**Table 2:** Show the classified instances results and J48 pruned tree assessment.

Classified instances						
Clusters	N = 281	Percent	Classified ratio			
			Diabetes Patient		Not Diabetes Patient	
0	138	49	227		54	
1	143	51				
**J48 Pruned tree**						
**Tested**		**GLU**	**BDPR**	**N = 281**	**TREE**	
					**Size**	**Leaves**
Positive		>101	No counts	191	7	4^b^
Negative		≤101	≤100	85		
Positive		>72	>100	3		
Negative		≤72	>100	2		

^b^N = total number of patients; GLU = glucose level; BDPR = blood pressure level.

**Figure 3:**
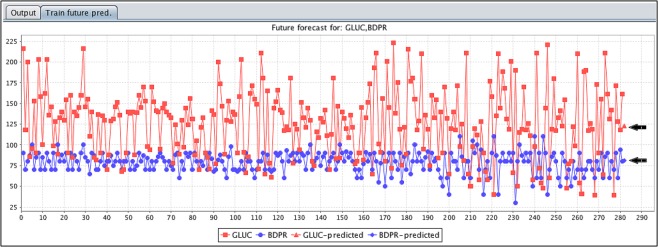
Show the future forecast prediction assessment done on the classification resulted attributes (Blood pressure and Glucose level).

Furthermore, the results obtained by the classification are used to predict future predictions by linear regression. “Figure 3” shows the future predictions for glucose and blood pressure properties as follows: If the blood pressure (BDPR > 80) and glucose level of diabetic patients reach (GLUC ≥ 125), the insulin injection will be high, which will lead to the death of diabetic patients. The black arrows in the figure mark the predictive evaluation of the two attributes.

### Cost/benefit matrix

**Figure 4:**
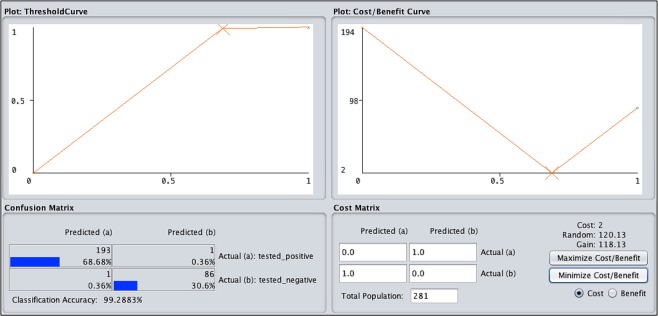
Show the cost/benefit assessment achieved by the decision tree J48 machine learning classifier into four parts as explained in the text.

“Figure 4” presents the cost/benefit assessment matrix for 281 diabetes patients datasets achieved by experiment measurements into four parts. The first part indicates the threshold plot curve ratio between the classified instances from 0 to 1 and the second part indicates the predicted confusion matrix between the tested positive and tested negative clusters with an accuracy of 99.28%. From 281 instances 68.68% classified as tested positive for the first prediction and 30.60% for the second prediction in the matrix. In addition, one instance with a ratio of (0.36%) classified as tested negative in both first and second prediction.

Moreover, the third part presents the classified instance cost/benefit plot graphically between the predicted clusters 0 and 1 from (0 to 193%) predicted instances and fourth part shows the cost matrix of two key attributes (glucose level and blood pressure) with the total gain of 118.13% and a random ratio of 120.13% in prediction instances of 281 diabetes patients.

## Discussion

In this study, the machine-learning technique is instigated on a data-mining platform with a dataset of 281 patients suffering from diabetes. The data are collected only from Pakistan for the assessment of diabetes mellitus prevalence by determining J48 classifier and linear regression to forecast the classification results on invasive and non-invasive medical attributes/variables. It includes the age (of the patient), gender (male and female), glucose level, body mass index, hypertension, diet (healthy and unhealthy), and utmost 100 other features to accurately measure the cost/benefit ratio of the predictive negative instance on classification results for medical specialists. Why the Pakistan region is significant^25^? Because it voluntarily enters in the developing countries, where the modern life necessitates, require with the enormous number of ratios and diabetes is the vastly spreading disease causing many other symptoms of cancer, blindness, and heart strokes.

The assessment has been launched on the data-mining platform (Weka) and the data set was divided into two parts for training and testing at a ratio of 20:80%. The 20 percent of the data was used to train the machine and measure the outcome whereas; the rest of 80 percent was used for testing analysis. In addition, a complete dataset of 281 patients was analyzed on the experimental mode of Weka for the assessment of decision tree classifiers namely J48 with an accuracy of (99.28%), J48 consolidation is (98.93%), J48 graft is (98.93%), and Hoeffding tree is (91.10%) respectively, also shown in “Table 3”. The J48 decision tree classification results show that the average accuracy is 99.28% and the error rate is 0.08%. In addition, the average Kappa was 0.98%, the true positive rate was 0.99%, the false positive rate was 0.08%, the precision, the recall rate, ROC and F-matrix were 0.99%, and the PRC and MCC were 0.98%.

**Table 3:** Show the classification result of four Decision-making classifiers and compares the utilized classifier with the recent author publication.

Algorithms	Acc%	Error rate	Kappa stats	Time taken	Data used	Year	Ref/worked by	
J48	98.38	Artificial dataset on 2017					^28^	O. Barrios
J48	99.28	0.01	0.98	0.08	Real-life	2018	This Paper^c^	
J48 consolidation	98.93	0.01	0.97	0.15				
J48 graft	98.93	0.01	0.97	0.02				
Hoeffding Tree	91.10	0.13	0.78	0.11				

^c^Acc = accuracy; % = percentage, Ref = reference.

The outcomes of the invasive and non-invasive medical features used in this study indicate that this assessment can accurately help to predict the patients of diabetes and pre-diabetes without necessitating any preliminary laboratory tests and equally useful for the medical specialists to analyze the cost/benefit on the tested negative patients for diabetes. In addition, the J48 decision rule generated during the assessment showed the main characteristics of diabetic patients were blood pressure and blood glucose levels. Therefore, Therefore, this study proposes a prediction that age is a potential and root variable, followed by blood pressure and blood sugar levels. These implementations are useful for low socioeconomic status regions around the globe, i.e. Africa, Asia, and other developing countries.

The cost-benefit principles span the key area of diabetes from the patients to analyze the record statistics of insulin level or with other treatment terminologies of diabetic patients. Put differently, cost-benefit analysis frequently finds quantification and traditionally includes all the valid factors of interest, rather than determining, reckoning and subtracting all negative ratios, called costs. The major difference between these two terms is whether the planned action is advisable or not for the patients. In Pakistan, diabetes is a rapidly growing vascular disease and is intimately associated with increased visual impairments with other subsets. The cost-benefit analysis can undoubtedly help the medical specialists and private doctors to analyze the tested negative ration of a patient with their medical stages. It works diligently with an accurate prediction of tested positive and tested negative ratios.

In the proposed method, cost/return has a good predicted percentage. From 281 instances, the positive rate of the predicted confusion matrix was 193 (68.68%) and the negative was 86 (30.60%). These two instances are classified as non-category for cost analysis with a forecast ratio of 0.36%. The cost/benefit analysis graph shows a clear threshold curve prediction for medical experts. The characteristics obtained by J48 decision-making algorithm are:

i. It handles discrete and continuous attributes correctly. The threshold is determined by C4.5 and is used to process continuous attributes. This value divides the data list into those whose attribute values are below the threshold and remain greater than or equal to the threshold.
ii. It also handles missing values in training data.
iii. After constructing the decision tree, J48 performs the appropriate pruning of the tree by pruning the branches that do not contribute to the leaf nodes.

The goal of this study is to utilize all decision-making classifiers (J48, J48 consolidation, J48 graft, and Hoeffding tree) with logistic regression assessment to identify and forecast diabetes mellitus prevalence. Similarly, the use of realistic health records collected from the seven principal hospitals of ‘Pakistan’, where the prevalence proportion of diabetes in men and women is high and explicitly mentioned in the literature. Hence, forming decision-making rules can screen the patients with diabetes mellitus and also mellitus can be controlled through organizing appropriate educational programs in developing countries that will help to govern the widespread growth of diabetes mellitus. The classification assessment of J48 proposed in this paper was set to compare with the most recent achievement from other authors as shown in table three, which clearly indicates J48 decision making classifiers have been successful in clinically meaningful research of this study.

The proposed study experiences two limitations. The first includes the training and test data partitioning of the Meta dataset, and the second is the time required to test the metadata for classification. If the metadata are analyzed on the same platform, the processing time can be further increased; it depends on the type of dataset, the number of seed inputs, and the number of experiments performed to achieve the desired result.

In conclusion, the proposed approach encourages and supports medical professionals to correctly assess the classifications of diabetes mellitus patients. In addition, future study can be conducted by utilizing this approach on Metadata. Particularly, the medical specialists can use the proposed approach to forecast diabetes mellitus patient’s attributes on the initial stages of apparent prevalence or on the initial stage of screening and diagnosing diabetes mellitus type 1, type 2 and gestational under the more constant decision-making phase.

## Methods

The Ethics committee of Yanshan University, Natural science foundation of China Hebei province, approved the study and all experiments procedures conformed to the Declaration of Helsinki. All participants provided written informed consent after all procedures were explained orally and in writing.

**Figure 5:**
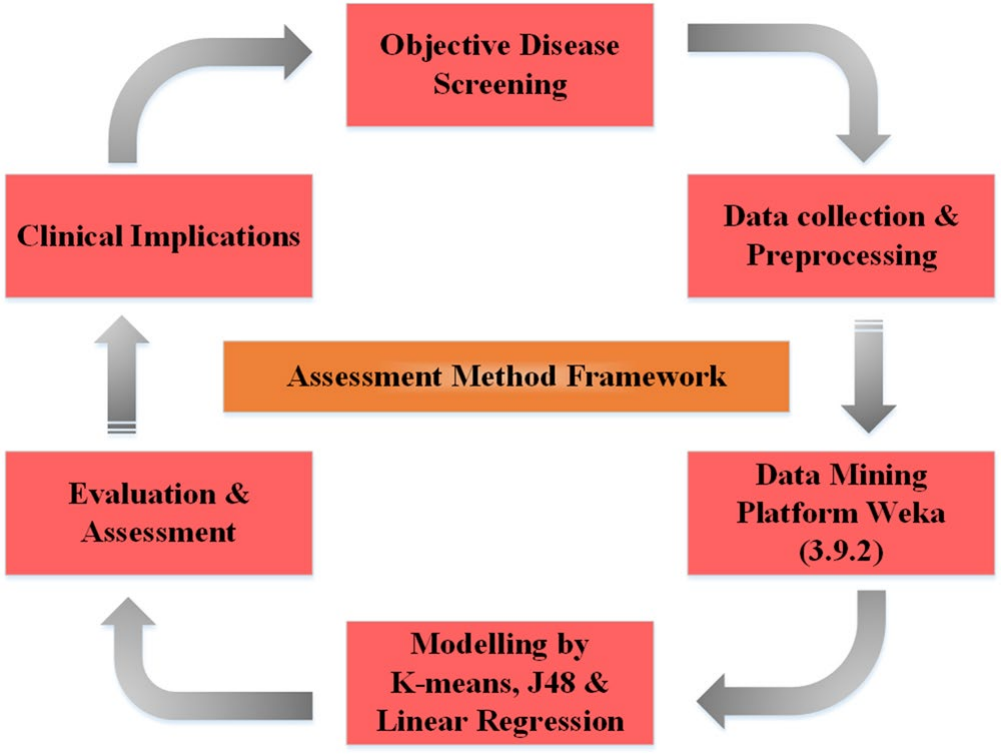
Show the assessment methodology framework into six steps as explained in the main text.

“Figure 5” displays the assessment framework used in this work for screening and cost benefit implications (performed in six steps) in diabetes patients. Initially, the real-life diabetes mellitus data was acquired and preprocessed afterward; the data was used for evaluation and assessment. Secondly, the updated plugins of machine learning J48 decision tree classifier were used on Weka version 3.9.2 “data mining platform” for classification measurements and assessment. Additionally, the linear regression method was employed on the results of machine learning classifiers to forecast the future predictions.

**Figure 6:**
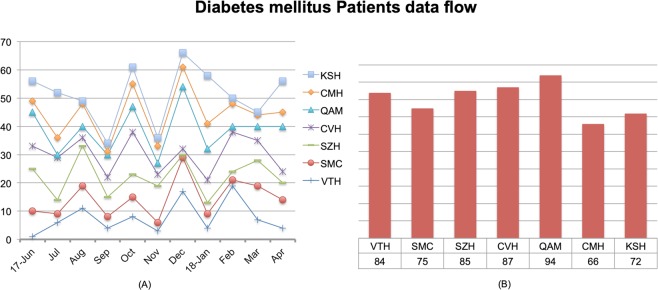
Show the diabetes data collection flow into two parts as described in the text.

The realistic dataset of 563 diabetes patients was collected by questionnaire design and verbal interviews from seven principal hospitals of Pakistan through time span of June 2017 to April 2018 with 108 medical features. “Figure 6” shows the tributaries namely with percentage of patients suffering from diabetes includes Victoria hospital (14.92%), Sharif medical college (13.32%), Sheikh Zaid hospital (15.09%), Civil hospital (15.45%), Quaid Azam medical college (16.69%), Central medical hospital (11.72%), and Khalid Saeed hospital (12.78%). The first part of figure shows the number of diabetes mellitus patients visited the mentioned hospitals by months and the second part show the total number of patients in each hospital graphically.

Out of 563 records, 282 records were removed from the dataset due to missing values in various attributes i.e. glucose level, patient’s occupation, last visit to the doctor, insulin injection intake, body mass index (BMI), work stress and many others out of 108 attributes. 281 records were analyzed for the model assessment of cost benefit analysis of diabetes patients. These records include the diabetes mellitus patients both male and female from age of (≥15) and (≤87), who was suffering other disease symptoms equally. The parameter selection for each attribute was analyzed carefully for the assessment as Age and gender represent demographic characteristics. Patients Glucose level (mmoI/L) has a relation with the age and diet. Family history of diabetes was defined as any family member previously diagnosed by a physician as diabetic or pre-diabetic (Yes = 1, No = 0). BMI was calculated as body weight divided by the square of height in meters and (BMI ≥ 25) was defined as overweight. Out of 108 attributes, other attributes were optimized at the initial stage by the values of 0 and 1 according to their mellitus relations. Numeric attributes were instantly changed to the nominal values presenting 0 as No and 1 as Yes by (3) to reduce the cognitive complexity of the dataset to properly conduct the assessment but carefully used the real values for the attributes like blood pressure, glucose and insulin to forecast the cost benefit predictions.

3 $${\rm{Values}}=({\rm{value}}-{\rm{x}})/{\rm{s}}$$

Data mining platform Waikato environment of knowledge analysis (Weka/v 3.9.2) was utilized for the preprocessing, classification assessment of cost benefit analysis, and forecast prediction of diabetes mellitus patients by updated plugins of machine learning algorithms K-means for clustering and J48 for classification^26^. In addition, linear regression was used on Weka for the future forecast assessment^27^. Initially, data was divided into 20/80 ratios in Weka by automatic filtering method, 20% was used as training dataset to train the machine, and 80% was utilized for initial classification evaluation to assign class attributes to final data sets for testing as positive and negative status of diabetes mellitus. Finally, the 100% dataset of 281 instances were utilized on the experimenter mode of Weka for the final assessment of classification and clustering. In addition, the generated results of classification were implemented for the forecast prediction analysis by linear regression. The advantage of using Weka is to avoid the over fitting and unnecessary complexity in the dataset.

K-means clustering algorithm is distance-based cluster and the distance measured by similarities in the cluster instances^20^. The K-means parameters selection was 100 for maximum canopies to hold in memory, minimum canopy density was set as 2.0, canopy pruning rate as 10000, canopy T_1_ as (−1.25), canopy T_2_ as (−1.0), functioning distance was set as Euclidean^20^, maximum iterations as 500, number of clusters as 2, execution slots as 1, and seeds value as 10.

The idea works behind K-means is invariably to define the K centroids for each cluster but these cluster centroids should be carefully placed in a scheming way because of the diverse locations produces inconsistent results. Therefore, the centroids should be prudently placed with the certain distance as much as possible from each other. After that it instantly seizes each point legitimately belonging to the dataset given and positively associate it to the most adjacent centroids. If there is no specific point remains than the first phase of the algorithm process is complete and the primary groupie is done. By this stage, it recalculates the K new centroids as barycenter of the clusters as resulted from the first phase. A new bound assuredly has to be done correctly after the K creative centroids between the points of same dataset and the most adjacent new centroid. For that the continuous loop generates to shift the specific location of K centroids on each necessary step and repeats until no centroid move anymore. This process executes as in (4–6).

4 $${S}_{i}^{(t)}=\{\forall j,\,1AjAk{X}_{p}:{\Vert {X}_{p}-{m}_{i}^{t}\Vert }^{2}\le {\Vert {X}_{p}-{m}_{j}^{t}\Vert }^{2}\forall j,1\le j\le k\}\forall j,1AjAk$$

5 $${{\rm{m}}}_{{\rm{i}}}^{t+1}=\frac{1}{|{{\rm{S}}}_{{\rm{i}}}^{({\rm{t}})}|}\sum _{{{\rm{x}}}_{{\rm{j}}}\in {{\rm{S}}}_{{\rm{i}}}^{({\rm{t}})}}\,{{\rm{X}}}_{{\rm{j}}}$$

6 $$J=\sum _{j=1}^{k}\,\sum _{i=1}^{n}\,{\Vert {x}_{i}^{(j)}-{c}_{j}\Vert }^{2}$$

Where n is the number of data points in the *i* clusters and k is the number of cluster centers and $\parallel {x}_{i}^{(j)}-{c}_{j}\parallel$ represents the Euclidean distance between ${x}_{i}^{(j)}$ and *c*_*j*_. Basically, the Kmean algorithm for the clustering is composed of the following steps on which it works further modeled as in (7, 8).

i. Place the K points into the considerable space as represented by the objects that are being clustered. These essential points sufficiently indicate the initial group of centroids.
ii. Properly assign each object to the group that undoubtedly possesses the most adjacent centroid.
iii. After the assigning all objects, recalculate the prominent position of K centroid.
iv. Repeat fiercely the second and third step until the centroids no able to move more significantly. This efficiently produces the possible separation of groups objects which the matric to be minimized can be accurately calculated.

7 $$argmi{n}_{{c}_{j}\in C}dist{({c}_{i},x)}^{2}$$

8 $${C}_{i}=\frac{1}{|{S}_{i}|}\sum _{{X}_{i}\in {S}_{i}}\,{X}_{i}$$

Decision tree classification helps to find out the vectors behave for the instance numbers^3,12,22,23^. After separation assessment of positive and negative clusters, this study adopted four decision-making classification algorithms with updated plugins in order to achieve the cost benefit classification for diabetes mellitus patients dataset namely J48, J48 consolidation, J48 graft, and Hoeffding tree. Finally, the J48 machine learning algorithms was utilized with 10-fold cross validation technique for the decision-making classification after the comparison assessment of four algorithms. In addition, the final classification results of J48 were used to forecast the predictions by linear regression on Weka. Essentially, J48 algorithm obtains the extension of ID3 and typically retains the additional formats for solving down the missing values, values ranges, and rules derivation. In addition, it is precisely an open source java implementation program of C4.5 algorithm in Weka (3.9.2), which includes three fundamental steps in our assessment model as:

i. In the specific case where the instance correctly belongs to a similar class, the tree represents the leaf that replaces the label with the corresponding class.
ii. The information is calculated for each attribute. At that considerable time, the gain information was being calculated by a test on attributes.
iii. The best branching attribute for decision tree assuredly found on the consistent basis of the present selection criterion.

The parameter selection for J48 was 100 as batch size with confidence interval of 0.25%, minimum number of objects and the decimal number places were set as 2, numbers of folds were set as 3, and error pruning as false with number of seeds equal to 1. The counting gain process would gently take the Entropy’s, where Y is calculated as (9, 10) and the total gain is calculated as in (11) to split the argument Y by value of J. Furthermore, the kappa stats for the classification accuracy measurements were analyzed as (12, 13).

9 $$Entropy(Y)=-\sum _{j=1}^{n}\,({Y}_{i})/|Y|.\,\mathrm{log}({Y}_{i})/|Y|$$

10 $$Entropy(J|Y)=(|{Y}_{j}|/|Y|).\,\mathrm{log}\,(|Y|/|Y|)$$

11 $${\rm{Gain}}\,({\rm{Y}},\,{\rm{J}})={\rm{Entropy}}\,({\rm{Y}}\mbox{--}{\rm{Entropy}}\,({\rm{J}}|{\rm{Y}}))$$

12 $$K=[P(A)-P(E)]/[1-P(E)]$$

13 $$P(E)=[(TP+FN)\ast (TP+FP)\ast (TN+FN)/{N}^{2}$$

The successful outcomes of J48 were implemented on linear regression to forecast the cost benefit assessment of decision tree attributes. Parameters for linear regression were set as 100 for batch size with attribute selection method M5, number of decimal places were set as 4 with ridge rate as (1.0E-8). In addition, linear regression works by using the values 0 and 1 along the logistic regression algorithm as (14), because the logistic regression deals with binary codes of values to accurate mapping of data items and has the ability to perform admirably the specific task using sigmoid function layers as followed in (15–17).

14 $$P=\alpha +{\beta }_{1}{X}_{1}+{\beta }_{2}{X}_{2}+\ldots +{\beta }_{m}{X}_{m}$$

15 $$\sigma (x)\frac{1}{1+{e}^{-x}}\in [0,\,1]$$

16 $${\Pr }(Y=+\,1|X) \sim \beta .X$$

17 $${\Pr }(Y=-\,1|X)=1-{\Pr }(Y=+\,1|X)$$

### Ethical approval and informed consent

Approval: We confirm that all experimental protocols were approved by Yanshan institute and has approved in method section of this paper. Accordance: All methods were carried out in accordance with the relevant guidelines and regulations. Informed consent: We confirm that informed consent was obtained from all participants and/or their legal guardian/s. Patient’s information: We confirm that informed consent to publish identifying information/images was obtained must be included in the methods section and we also identify that images/video/details about patient’s, has been removed from the paper due to policy restrictions.

## Supplementary information

Supplementary information accompanies this paper at https://doi.org/10.1038/s41598-019-46631-9.

## Data Availability

All data utilized in this study is available as a supplementary information file.
