# Supplementary material for: A hybrid Forecast Cost Benefit Classification of diabetes mellitus prevalence based on epidemiological study on Real-life patient’s data
Source: Sci Rep. 2019 Jul 12;9:10103. doi: 10.1038/s41598-019-46631-9 (PMC6626127; doi:10.1038/s41598-019-46631-9)
Supplement: Supplementary file 1 — Supplementary information accompanies this paper at https://doi.org/10.1038/s41598-019-46631-9. [file 41598_2019_46631_MOESM1_ESM.pdf]

A hybrid Forecast Cost Benefit Classification of diabetes mellitus prevalence based on epidemiological study on Real-life patient's data

Muhammad Noman SOHAIL[1*], Ren JIADONG[1], Musa Muhammad UBA[1], Muhammad IRSHAD[1], Wasim IQBAL[2], Jehangir ARSHAD[3], Antony Verghese JOHN[4]

[1] Dept. Of Information Sciences and Technology. Yanshan University, Hebei, China

[2] Dept. Of Economics and Management. Yanshan University, Hebei, China

[3] Dept. Of Computer Sciences and Technology. Comsat University, Islamabad, Pakistan

[4] Dept. Of Hotel Management, American Hotel and Lodging Association, New York, United States of America

[*]Corresponding Author
Name: Muhammad Noman SOHAIL
Yanshan University, Hebei, China.
Email: mn.sohail@stumail.ysu.edu.cn
ORCID: 0000-0001-6914-5505
Phone: +8615032370085

Academic Research Questionnaire on Diabetic patients

School of Information Sciences & Technology YANSHAN University, Qinhuangdao City

P.R China

Dear Participant:

It's a pleasure to introduce myself as a student in above-mentioned academic institute as Doctorate of Philosophy Researcher, who's under going with a research in field of "Diabetic Data Analysis". To go on with this research, please I need your assistance in the process of answering these questions. It will be really appreciated and can be helpful for me to carry on the research with good scope. Please feel free to answer all questions, all information's will be highly confidential and strictly for academic research purpose. Been selected as the participant in this study, at any point of time you may wish to withdrawn freely with no reservation what so ever.

The main goal for conducting the exercise is to collect primary data in order to mine the relationship between multi-dimensional physiological parameters and diseases to come-up with valuable medical information for supporting medical decisions making.

Noman Sohail

Researcher

Questions:

1. Participant's Age...

2. Weight…………………………………………………………………….............

3. Body mass index……………………………………………………………

4. Type……………………………………………………………………………..

5. Occupation.…………………………………………………………….............

6. Sex: Tick ( ) only one Male Female

7. Your Diet (Food Type eaten).……………………………………………........

8. Marital Status: Tick ( ) only one Married Separated Widowed Single Divorced

9. Residential Suburb: Tick ( ) only one City Town Village

10. Level of Education: Tick ( ) only one Not-go-to-school Primary-school High-school Collage/university Post-graduate Islamic- school

11. Please indicate below, the diabetes condition(s) you have: Tick ( ) only one

Insulin dependent Non-insulin dependent

Gestational diabetes Other chronic condition Specify........................

In general, patients suffering from any of category may encounter some or all of the following symptoms: Tick ( ) only one

12. Excessive thirst? Yes or No

13. Frequent Urination? Yes or No

14. Unexplained weight loss or gain Yes or No

15. Flulike symptoms, weakness / fatigue Yes or No

16. Blurred vision Yes or No

17. Irritability? Yes or No

18. Slow healing cut or bruises Yes or No

19. Tingling or loss of feeling in hands or feet? Yes or No

20. Recurring infections of gum or skin Yes or No

21. Recurring vaginal bladder infection Yes or No

22. Please if there is any symptoms apart from above indicated specify?.............

Blood sugar level may drop for many reasons:

23. Skipping meal Yes or No

24. Exercising longer or more strenuously than normal Yes or No

25. Not adjusting your medication to accommodate changes Yes or No

Do you experience any of the following symptoms of hypoglycemia varying depending on how your blood sugar level drops?

26. Sweating Yes or No

27. Shakiness Yes or No

28. Visual disturbance Yes or No

29. Weakness Yes or No

30. Hunger Yes or No

31. Dizziness Yes or No

32. Nervousness Yes or No

33. Headache Yes or No

34. Fast Heartbeat Yes or No

35. Irritability Yes or No

36. Nausea Yes or No

37. Cold, clammy skin Yes or No

38. Slurred speech Yes or No

39. Drunken like behavior Yes or No

40. Drowsiness Yes or No

41. Confusion Yes or No

42. Convulsion Yes or No

Do you experience any of the following symptoms of Diabetic hyperosmolar syndrome (DHS)? High blood sugar level

43. Excessive thirst Yes or No

44. Increased in urination Yes or No

45. Weakness Yes or No

46. Leg cramps Yes or No

47. Confusion Yes or No

48. Rapid pulse Yes or No

49. Coma Yes or No

Do you experience any of the following symptoms of Diabetic ketoacidosis (DKA)? Increase in blood acid

50. Deep, rapid breathing Yes or No

51. Sweet, fruity smell on your breath Yes or No

52. Loss of appetite Yes or No

53. Nausea Yes or No

54. Fever Yes or No

55. Stomach pain Yes or No

56. Weight loss Yes or No

57. Weakness Yes or No

58. Fatigue Yes or No

59. Confusion Yes or No

60. Drowsiness Yes or No

Do you experience any of the following symptoms of Nephropathy? Kidney damage

61. Swelling of the ankles, feet and hands Yes or No

62. Shortness of breath Yes or No

63. High blood pressure Yes or No

64. Confusion or difficulty concentration Yes or No

65. Poor appetite Yes or No

66. Nausea Yes or No

67. Vomiting Yes or No

68. Dry, itchy skin Yes or No

69. Fatigue Yes or No

Do you experience any of the following symptoms of Retinopathy? Eye damage

70. Spiders, cobwebs or tiny specks floating in an eye Yes or No

71. A gray shadow in your vision Yes or No

72. Blurred words while reading Yes or No

73. A dark or empty spot in the center of your vision Yes or No

74. Dark streaks or a red that blocks vision Yes or No

75. Eye pain Yes or No

76. Flashes of light or ring around objects Yes or No

77. Straight line that appears distorted Yes or No

78. Vision loss Yes or No

Do you experience any of the following symptoms of cardiovascular problems? Heart/chest related diseases

79. Shortness of breath Yes or No

80. Pain in your chest, jaw or arm Yes or No

81. Fatigue and weakness Yes or No

82. Swelling (edema) Yes or No

83. Lightheadedness Yes or No

84. Rapid or irregular heartbeats Yes or No

85. Excessive perspiration Yes or No

Over production of hormone is called **Hyperthyroidism** and may cause High blood pressure / Heart rate. Did you experience any of its symptoms?

86. Nervousness Yes or No

87. Heat intolerance Yes or No

88. Palpitations Yes or No

89. Tremor Yes or No

90. Fatigue Yes or No

91. Weight loss Yes or No

92. Prominent eyes (exophthalmoses) Yes or No

93. Enlarge thyroid or absence of nodules on your thyroid Yes or No

Under production of hormone is called **Hypothyroidism** and may cause High blood pressure. Did you experience any of its symptoms?

94. Cold intolerance Yes or No

95. Fatigue Yes or No

96. Slowing in body functions Yes or No

97. Weight gain Yes or No

98. Coarse skin Yes or No

99. Low, husky voice Yes or No

100. Puffiness about your eyes, legs and hand Yes or No

Medications / Medical Care

101. In the past week did you take pills for diabetes? Yes or No

If yes, please specify the name(s) of the diabetes pills you took………………....

102. In the past week did you get insulin injections? Yes or No

103. In the past week did you take pills for high blood pressure? Yes or □ No

If yes, please specify the name(s) of the blood pressure pills you took…..……...

104. In the past week did you take pills for cholesterol? Yes or □ No

If yes, please specify the name(s) of the cholesterol pills you took…...………...

105. When you visit your doctor last?..

106. In the past 6 months, how many times did you visit a physician?.....................

107. How many times did the doctor examine your feet in the last 6 month?.............

108. Would you engage in exercise(s) in support of your fitness? Tick ( ) only one

Stretching or strengthening exercises

Walk for exercise Bicycling (Stationary or Bike)

other aerobic exercise equipment's specified?..

Thank you for your help!

Data set Attributes

The questionnaire data has converted in to specific attributes as accurately shown in the Table 1.

Table 1: *Dataset attributes used in the successful experiment after efficiently converting from hard copy format to soft copy format*

**Attributes**	**Description**	**Reports**	**Type**
Patients	Male and Female		
AGE	Patient age	Above 15 and less than 90	Numeric
WGT	Weight	Patient weight in Kg's	Numeric
GLU	Glucose	Values in numbers	Numeric
BDPR	Blood pressure	Values in numbers	Numeric
MASS	Mass	Values in numbers	Numeric
EXCT	Excessive thirst	Values: 0= -ve and 1= +ve	Numeric
DTYP	Diabetes type	NID, IND, GTD	Nominal
FQUR	Frequent urination	Values: 0= -ve and 1= +ve	Numeric
WGLG	Weight loss/gain	Values: 0= -ve and 1= +ve	Numeric
SPTM	Flulike symptoms	Values: 0= -ve and 1= +ve	Numeric
VSIN	Blurred vision	Values: 0= -ve and 1= +ve	Numeric
IRTB	Irresistibility	Values: 0= -ve and 1= +ve	Numeric
HEAL	Slow healing	Values: 0= -ve and 1= +ve	Numeric
TNGL	Tingling's	Values: 0= -ve and 1= +ve	Numeric
SKIF	Skin infection	Values: 0= -ve and 1= +ve	Numeric
VGIF	Vegetal infection	Values: 0= -ve and 1= +ve	Numeric
SWET	Sweating	Values: 0= -ve and 1= +ve	Numeric
SHIV	Shivering	Values: 0= -ve and 1= +ve	Numeric
VSBC	Visual disturbance	Values: 0= -ve and 1= +ve	Numeric
WKNS	Weakness	Values: 0= -ve and 1= +ve	Numeric

HNGR	Hunger	Values: 0= -ve and 1= +ve	Numeric
DZNS	Dizziness	Values: 0= -ve and 1= +ve	Numeric
NVUS	Nervousness	Values: 0= -ve and 1= +ve	Numeric
HDHE	Headache	Values: 0= -ve and 1= +ve	Numeric
FHBT	Fast heart beat	Values: 0= -ve and 1= +ve	Numeric
IRST	Irresistibility	Values: 0= -ve and 1= +ve	Numeric
NUSA	Nausea	Values: 0= -ve and 1= +ve	Numeric
CMSK	Clammy skin	Values: 0= -ve and 1= +ve	Numeric
SRSP	Slurred speech	Values: 0= -ve and 1= +ve	Numeric
DRHB	Drunken behavior	Values: 0= -ve and 1= +ve	Numeric
DRSN	Drowsiness	Values: 0= -ve and 1= +ve	Numeric
CFSN	Confusion behavior	Values: 0= -ve and 1= +ve	Numeric
CNVL	Convulsion	Values: 0= -ve and 1= +ve	Numeric
INUR	Increased urination	Values: 0= -ve and 1= +ve	Numeric
LGCP	Leg cramp	Values: 0= -ve and 1= +ve	Numeric
RPDP	Rapid pulse	Values: 0= -ve and 1= +ve	Numeric
COMA	Coma	Values: 0= -ve and 1= +ve	Numeric
DPBT	Deep rapid breath	Values: 0= -ve and 1= +ve	Numeric
BTSM	Breath smell	Values: 0= -ve and 1= +ve	Numeric
LOAP	Loss of appetite	Values: 0= -ve and 1= +ve	Numeric
FEVR	Fever	Values: 0= -ve and 1= +ve	Numeric
STPN	Stomachache	Values: 0= -ve and 1= +ve	Numeric
WGHL	Weight loss	Values: 0= -ve and 1= +ve	Numeric
FATI	Fatigue	Values: 0= -ve and 1= +ve	Numeric
DRWS	Drowsiness	Values: 0= -ve and 1= +ve	Numeric
BDSM	Body smelling	Values: 0= -ve and 1= +ve	Numeric

SHBE	Breath shortness	Values: 0= -ve and 1= +ve	Numeric
HBRP	High blood pressure	Values: 0= -ve and 1= +ve	Numeric
CNPB	Concentration	Values: 0= -ve and 1= +ve	Numeric
PAPT	Poor appetite	Values: 0= -ve and 1= +ve	Numeric
VOMT	Vomiting	Values: 0= -ve and 1= +ve	Numeric
DISN	Dry, itchy skin	Values: 0= -ve and 1= +ve	Numeric
SPCW	Spider cobwebs	Values: 0= -ve and 1= +ve	Numeric
GRSH	Vision shadow	Values: 0= -ve and 1= +ve	Numeric
BLUS	Blurred vision	Values: 0= -ve and 1= +ve	Numeric
DRVS	Empty spot vision	Values: 0= -ve and 1= +ve	Numeric
RDVS	Red vision	Values: 0= -ve and 1= +ve	Numeric
EYPN	Eye pain	Values: 0= -ve and 1= +ve	Numeric
LGFS	Light flashes	Values: 0= -ve and 1= +ve	Numeric
STRL	Straight line vision	Values: 0= -ve and 1= +ve	Numeric
VSLS	Vision loss	Values: 0= -ve and 1= +ve	Numeric
BDPN	Jaws pain	Values: 0= -ve and 1= +ve	Numeric
PRPT	Perspiration	Values: 0= -ve and 1= +ve	Numeric
HTIT	Heat intolerance	Values: 0= -ve and 1= +ve	Numeric
PLTN	Palpitation	Values: 0= -ve and 1= +ve	Numeric
TRMR	Tumor	Values: 0= -ve and 1= +ve	Numeric
PREY	Prominent eyes	Values: 0= -ve and 1= +ve	Numeric
CINT	Cold intolerance	Values: 0= -ve and 1= +ve	Numeric
SBFT	Slow body function	Values: 0= -ve and 1= +ve	Numeric
WHTG	Weight gain	Values: 0= -ve and 1= +ve	Numeric
CRSK	Course skin	Values: 0= -ve and 1= +ve	Numeric
HKSK	Husky skin	Values: 0= -ve and 1= +ve	Numeric

BDPF	Body puffiness	Values: 0= -ve and 1= +ve	Numeric
INIJ	Insulin injections	Values: 0= -ve and 1= +ve	Numeric
CHTP	Cholesterol pills taken	Values: 0= -ve and 1= +ve	Numeric
LDVD	Last time doctor visit in days	Values in numbers	Numeric
VDLM	Visit doctor in last six months	Values in numbers	Numeric
PHEX	Any medical checkup in last six months	Values in numbers	Numeric
Class	Diagnosis of Diabetes	Values: T_P and T_N	Nominal *

* The accurate table divinely reveals the comprehensive list of specific attributes, which we have properly used for the experiment purpose by training the modern machine and testing the specific methodology. NID= non-insulin dependent (Type 1 diabetes); IND= insulin dependent (Type 2 diabetes); GTD= gestational diabetes; T_P= tested positive; T_F= tested negative.

Data Set

"AGE","GLUC","BDPR","MASS","WGT","EXCT","FQUR","WGLG","SPTM","VSIN","IRTB","HEAL","TNGL","SKIF","VGIF","SWET","SHIV","VSBC","WKNS","HNGR","DZNS","NVUS","HDHE","FHBT","IRST","NUSA","CMSK","SRSP","DRBH","DRSN","CFSN","CNVL","INUR","WEKN","LGCP","CONF","RPDP","COMA","DPBT","BTSM","LOAP","NAUS","FEVR","STPN","WGHL","WAKN","FATI","CONI","DRWS","BDSM","SHBE","HBRP","CNPB","PAPT","NSEA","VOMT","DISN","FTGU","SPCW","GRSH","BLUS","DRVS","RDVS","EYPN","LGFS","STRL","VSLS","BTHP","BDPN","FGWK","SWLN","LGCN","PRPT","NVSN","HTIT","PLTN","TRMR","FATU","WEHT","PREY","THRD","CINT","FAUE","SBFT","WHTG","CRSK","HKSK","BDPF","INIJ","CHTP","LDVD","VDLM","PHEX","DTYP","class"

"58","216","90","19.9","61","0","1","0","0","0","0","0","0","0","0","0","0","0","0","0","0","0","0","0","0","0","0","0","0","0","0","1","0","0","0","0","0","0","1","0","0","0","1","0","0","0","0","0","1","0","1","0","0","0","0","0","0","0","0","0","0","0","0","0","0","0","0","0","1","0","0","0","0","1","0","0","0","0","0","0","0","0","0","0","0","0","0","28","3","1","NID","tested_positive"

"33","118","70","22.5","54","0","1","0","0","0","1","1","0","0","0","1","1","0","1","1","1","0","1","1","1","0","1","0","0","0","0","0","0","1","0","0","0","0","0","0","0","0","0","0","1","0","0","0

","0","0","0","0","0","0","0","1","0","0","0","0","0","0","0","0","0","0","0","0","0","0","0","0
","0","0","0","0","0","1","0","0","0","0","0","0","0","0","0","0","0","30","3","2","NID","tested_p
ositive"

"20","200","80","21","54","1","1","0","0","0","0","1","0","0","0","1","1","0","0","1","1","0","1",
"0","0","0","1","0","0","0","0","0","1","0","0","0","0","0","0","0","1","0","1","0","0","0","0","0",
"0","0","0","0","0","1","0","0","1","0","0","0","0","0","0","0","0","0","0","0","0","0","0","0","0",
"0","0","0","0","0","0","0","0","0","0","0","0","0","0","0","0","0","21","3","0","NID","tested_po
sitive"

"56","86","87","24","78","1","1","1","1","0","0","0","1","1","1","1","1","0","1","1","1","0","1","
0","0","0","1","1","0","1","0","0","1","1","1","0","0","0","1","0","1","1","1","0","1","1","0","0","
0","1","1","1","0","1","1","1","1","1","0","0","0","0","0","0","0","0","1","0","1","1","0","0","
0","0","1","0","1","1","0","0","0","1","1","0","0","0","0","0","0","42","4","1","NID","tested_neg
ative"

"57","96","100","20","64","1","1","0","1","0","1","1","1","0","1","1","1","0","0","1","1","1","0",
"0","0","0","1","1","0","0","1","0","1","1","1","0","0","0","0","0","1","1","0","1","1","0","0","0",
"1","1","0","1","0","1","1","1","1","1","0","0","0","0","0","1","1","0","0","1","1","1","1","0","0",
"0","1","1","0","1","0","0","0","0","1","1","0","0","0","0","0","0","28","3","2","NID","tested_ne
gative"

"35","153","70","19","65","0","1","0","0","0","0","1","0","0","0","1","0","0","1","1","0","0","0",
"0","0","0","0","0","0","0","0","0","1","0","1","0","0","0","0","0","0","0","0","0","0","0","0","0",
"0","0","0","0","0","0","0","0","1","0","0","0","0","0","0","0","0","0","0","0","0","1","0","0",
"0","0","0","0","0","0","0","0","0","0","0","0","0","0","0","0","0","7","8","5","NID","tested_posi
tive"

"57","90","85","17","49","0","1","1","1","0","0","1","0","1","0","1","1","0","1","1","1","0","0","
1","0","0","0","1","0","1","1","0","1","1","1","0","0","0","0","0","1","1","1","0","1","1","1","0","
0","0","0","1","0","1","1","1","0","0","0","0","0","0","0","0","0","0","0","1","0","1","0","0","0","
0","0","1","0","1","1","0","0","0","1","0","0","0","0","0","0","0","28","8","3","NID","tested_neg
ative"

"70","203","90","30","82","1","1","1","1","1","0","0","1","1","1","1","1","1","1","0","1","1","0",
"0","0","1","0","0","0","0","0","0","1","1","1","0","0","0","0","0","1","0","0","0","0","0","0","0",
"0","0","0","0","0","1","0","0","0","0","0","0","0","0","0","0","1","0","0","0","0","1","0","0","0",
"0","0","0","0","0","0","0","0","0","1","0","0","0","1","0","0","0","21","0","0","NID","tested_po
sitive"

"40","158","70","18","60","1","1","0","0","0","0","0","0","1","0","0","0","0","0","0","0","0","0",

"0","0","0","0","0","0","0","0","0","1","0","0","0","0","0","0","0","0","0","0","0","0","0","0",
"0","0","0","0","0","0","0","0","0","0","0","0","0","0","0","0","0","0","0","0","0","0","0","0",
"0","0","0","0","0","0","0","0","0","0","0","0","0","0","0","0","0","330","0","0","NID","tested_p
ositive"

"67","100","85","23","68","1","1","0","1","0","1","1","1","0","0","1","1","1","0","1","1","0","0",
"1","0","0","1","1","0","0","0","0","1","0","0","0","0","0","0","0","0","0","1","1","0","1","0","0",
"0","1","0","1","0","0","0","0","1","0","0","0","0","0","0","0","0","0","0","0","1","1","0","0",
"0","1","1","0","0","0","0","1","0","1","0","0","0","0","0","0","0","30","5","2","NID","tested_ne
gative"

"43","162","70","21.5","72","0","1","0","1","0","0","1","1","0","0","0","0","0","0","0","0","0","0
","0","0","0","1","0","0","0","0","0","1","1","0","0","0","0","0","0","1","1","1","0","1","1","0","0
","0","0","0","0","1","1","1","0","0","0","0","0","0","0","1","1","0","0","0","0","1","1","0","0","0
","0","0","1","0","0","1","0","0","0","0","0","0","0","0","0","0","0","28","4","2","NID","tested_p
ositive"

"69","203","90","23","72","1","1","1","1","1","0","1","1","1","0","1","1","1","1","1","1","1","1",
"1","1","1","1","1","0","0","1","0","1","1","1","1","1","0","1","0","1","1","1","0","1","1","1","0",
"0","1","1","1","0","1","1","1","0","1","0","0","0","0","0","0","1","0","0","1","1","1","1","0","0",
"1","1","1","0","1","1","0","0","1","1","1","0","0","1","0","0","0","14","6","6","NID","tested_po
sitive"

"42","136","80","18","62","1","1","0","1","0","0","0","0","0","0","1","0","0","1","1","0","0","0",
"0","0","1","0","0","0","0","0","0","1","1","0","0","0","0","0","0","0","1","0","0","0","1","0","0",
"0","0","0","1","0","0","1","0","0","0","0","0","0","0","0","0","0","0","0","0","0","1","0","0","0",
"0","0","0","0","0","0","0","0","0","0","0","0","0","0","0","0","0","60","2","0","NID","tested_po
sitive"

"28","146","70","18","49","0","1","1","1","0","0","0","0","0","0","1","0","0","1","1","0","0","0",
"0","0","0","0","0","0","0","0","0","1","1","0","0","0","0","0","1","1","0","0","0","1","1","0","0",
"0","0","0","0","0","1","0","0","0","0","0","0","0","0","0","0","0","0","0","0","0","1","0","0","0",
"0","0","0","0","0","1","0","0","0","1","1","0","0","0","0","0","0","42","2","0","NID","tested_po
sitive"

"36","99","90","28","74","1","1","0","1","1","0","0","0","0","0","1","0","1","1","1","0","0","0","
0","0","0","0","0","0","0","0","0","1","1","1","0","0","0","1","1","1","1","1","0","1","0","0","0","
0","1","1","1","0","1","0","0","0","0","0","1","0","0","0","0","0","0","0","1","1","1","1","0","1","
0","1","1","0","0","0","0","0","0","1","1","0","0","0","0","0","0","56","3","0","NID","tested_neg
ative"

"21","128","70","17","42","1","1","0","0","0","0","0","0","0","0","1","0","0","0","1","1","0","1",
"0","0","0","0","0","0","0","0","0","1","1","0","0","0","0","0","0","1","0","0","0","0","1","0","0",
"0","0","0","0","0","1","0","0","0","0","0","0","0","0","0","0","0","0","0","0","0","0","0","0","0",
"0","0","0","0","0","0","0","0","0","0","1","0","0","0","0","0","0","56","1","0","NID","tested_po
sitive"

"45","89","100","26","67","1","1","1","0","1","0","0","0","1","0","1","0","1","1","1","0","0","0",
"0","0","0","0","0","0","0","0","0","1","1","0","0","0","0","0","0","1","0","0","0","1","0","0","0",
"0","0","0","1","0","1","0","0","0","0","0","0","0","0","0","0","0","0","0","0","0","1","0","0","0",
"0","0","0","0","1","0","0","0","0","1","1","0","0","0","0","0","0","84","2","0","NID","tested_ne
gative"

"33","133","80","19","58","1","1","1","1","1","0","0","0","0","0","1","0","1","1","1","0","0","0",
"0","0","0","0","0","0","0","0","0","1","1","1","0","0","0","0","0","1","0","0","1","1","1","1","0",
"0","0","0","1","0","1","0","0","0","1","0","0","1","0","0","0","0","0","0","0","1","1","0","0","1",
"0","0","1","0","0","1","0","0","0","1","1","0","0","0","0","0","0","56","2","0","NID","tested_po
sitive"

"71","140","88","20","65","1","1","1","1","1","0","0","0","1","0","1","0","1","1","1","0","0","0",
"0","0","0","0","0","0","0","0","0","1","1","1","0","0","0","0","0","1","0","0","0","1","1","1","0",
"0","0","0","1","0","1","0","0","0","1","0","0","0","0","0","0","0","0","0","0","0","1","0","0","0",
"0","0","0","0","1","1","0","0","0","1","1","0","0","0","0","0","0","60","2","0","NID","tested_po
sitive"

"65","130","80","21","72","1","1","1","0","1","0","1","1","1","1","1","1","1","1","1","1","0","1",
"0","0","0","0","0","0","0","0","0","1","1","0","0","0","0","0","0","1","0","0","0","1","1","1","0",
"0","0","0","1","0","1","0","0","0","1","0","1","0","0","0","0","0","0","0","0","0","1","0","0","1",
"0","0","0","0","1","1","0","0","0","1","1","0","0","0","0","0","0","28","3","0","NID","tested_po
sitive"

"40","154","90","27","85","1","1","1","0","0","0","0","0","0","0","1","0","1","1","1","1","0","0",
"0","0","0","0","0","0","0","0","0","1","1","1","0","0","0","0","0","1","0","0","0","1","1","0","0",
"0","1","1","1","0","1","0","0","0","0","0","1","0","0","0","0","0","0","0","1","1","1","1","0","0",
"0","0","1","0","1","0","0","0","1","1","1","1","0","0","0","0","1","56","3","0","NID","tested_po
sitive"

"72","90","70","22","58","1","1","1","0","0","0","0","1","0","0","1","1","1","1","1","0","1","0","
0","0","0","1","1","0","0","1","0","1","1","0","1","0","0","0","0","0","0","1","0","1","1","1","1","
0","1","0","0","1","0","0","0","1","1","0","0","0","0","0","1","0","0","0","0","0","1","1","0","0","
0","0","1","0","1","0","0","0","0","1","0","0","0","0","0","0","0","90","2","0","NID","tested_neg

ative"

"75","160","90","17.5","71","1","1","0","0","1","0","1","1","0","0","1","1","1","1","1","1","0","1
","1","0","1","1","0","0","0","0","0","1","1","1","0","0","0","0","0","1","1","1","1","1","1","1","0
","0","1","0","1","0","1","1","1","1","1","0","1","0","0","0","1","1","0","0","0","1","1","1","0","0
","0","0","0","0","1","0","0","0","0","1","0","0","0","0","0","0","0","7","4","4","NID","tested_po
sitive"

"55","85","70","22","82","1","1","0","0","0","0","1","1","0","0","1","1","0","1","1","1","0","1","
1","0","0","1","0","0","0","1","0","1","1","0","1","0","0","0","0","0","0","0","0","0","1","1","1","
0","1","0","0","0","0","0","0","1","1","0","0","0","0","0","1","0","0","0","0","1","1","1","0","0","
0","0","0","0","1","0","0","0","0","1","0","0","0","0","0","0","0","90","1","0","NID","tested_neg
ative"

"32","140","87","27","65","1","1","0","1","0","0","0","1","1","1","0","0","0","1","1","1","0","1",
"0","0","0","1","0","0","0","0","0","1","1","1","0","0","0","0","0","0","0","1","0","0","1","1","0",
"0","1","0","1","0","0","0","1","0","1","0","0","0","0","0","0","0","0","0","0","0","1","1","0","0",
"0","0","1","0","1","0","0","0","0","1","1","0","0","0","0","0","0","30","4","2","GTD","tested_po
sitive"

"45","135","80","22","75","1","1","1","0","0","0","0","0","0","0","0","0","0","0","0","0","0","0",
"0","0","0","1","0","0","0","0","0","1","1","1","0","0","0","0","0","0","1","0","0","0","1","1","0",
"0","1","0","0","0","0","1","0","0","1","0","0","0","0","0","0","0","0","0","0","0","1","1","0","0",
"0","0","0","0","1","0","0","0","0","1","0","1","0","0","0","0","1","14","6","6","GTD","tested_po
sitive"

"39","145","70","19","65","1","1","0","1","1","0","0","1","1","1","1","1","1","1","1","1","1","1",
"1","0","1","1","1","1","0","1","1","1","1","1","1","1","1","0","0","0","1","1","1","0","1","1","1",
"1","1","0","0","1","0","1","1","1","1","0","0","1","0","0","1","1","0","0","0","1","1","1","0","0",
"1","1","1","0","1","1","1","1","0","1","0","0","0","1","0","0","0","42","3","1","NID","tested_po
sitive"

"65","160","90","25","69","1","1","0","1","1","0","0","1","0","0","0","0","1","1","1","1","0","0",
"0","0","1","0","0","0","0","0","0","1","1","0","0","0","0","0","0","0","1","0","0","0","1","1","0",
"0","0","0","1","0","0","1","0","1","1","0","0","1","0","0","0","1","1","1","0","0","1","0","0","0",
"0","0","1","0","1","0","0","0","0","1","0","0","0","0","0","0","0","7","5","0","NID","tested_posi
tive"

"70","216","100","20","58","1","1","0","1","1","1","1","1","0","0","1","1","1","1","1","1","0","0
","0","1","0","1","0","0","0","0","0","1","1","0","0","0","0","0","0","0","0","0","0","0","1","1","0
","0","1","0","1","0","0","0","0","1","1","0","0","0","0","0","1","0","0","0","0","0","1","1","0","0

","0","0","1","0","1","0","0","0","0","1","1","0","0","0","0","0","0","14","4","4","NID","tested_p
ositive"

"62","146","85","29","89","0","0","0","0","0","0","0","0","0","0","0","0","0","1","0","0","0","0",
"0","0","0","0","0","0","0","0","0","0","1","1","0","0","0","0","1","0","0","0","0","1","1","0","0",
"0","0","0","0","0","0","0","0","0","0","0","0","0","0","0","0","0","0","0","0","1","1","0","0","0",
"0","0","0","0","0","0","0","0","0","0","0","0","0","0","0","0","0","49","3","0","NID","tested_po
sitive"

"65","155","80","20","60","0","1","0","0","1","0","0","0","0","0","0","0","1","1","0","0","0","0",
"0","0","0","0","0","0","0","0","0","0","0","1","0","0","0","0","0","0","0","0","0","0","0","1","0",
"0","0","0","0","0","0","0","0","0","1","1","0","0","0","0","0","0","0","0","0","0","0","0","0","0",
"0","0","0","0","1","0","0","0","0","0","1","0","0","0","0","0","0","7","2","0","NID","tested_posi
tive"

"61","110","65","20","62","0","1","1","1","1","1","0","0","1","0","0","0","1","1","0","0","0","0",
"1","1","0","0","0","0","0","0","0","1","1","1","0","0","0","0","0","0","1","0","0","1","1","1","0",
"0","0","0","0","0","0","1","0","0","1","1","0","0","0","0","1","0","0","1","0","0","1","0","0","0",
"0","1","0","0","1","1","1","0","0","1","1","0","0","0","0","0","0","90","2","0","NID","tested_po
sitive"

"65","140","90","23.5","80","0","1","1","0","0","0","0","0","0","0","0","0","0","0","0","0","0","1
","0","0","0","0","0","0","0","0","0","1","0","1","0","0","0","0","0","0","0","0","0","1","1","1","0
","0","0","0","1","0","0","0","0","0","1","0","0","0","0","0","0","0","0","0","0","0","1","0","0","0
","0","0","0","0","1","1","0","0","0","1","1","0","0","0","0","1","0","28","2","0","IND","tested_p
ositive"

"62","85","87","26","70","0","0","0","0","0","0","0","1","0","0","0","0","0","0","0","0","1","0","
0","0","1","0","0","0","0","0","0","0","1","1","0","0","0","0","0","0","0","0","0","0","1","0","0","
0","0","0","1","0","0","0","0","0","0","0","0","0","0","0","0","0","0","0","0","0","0","0","0","0","
0","0","0","0","0","0","0","0","0","0","0","0","0","0","0","0","0","35","4","0","NID","tested_neg
ative"

"44","82","70","21","57","0","0","0","0","0","0","0","1","0","0","0","0","1","0","0","0","0","
0","0","0","0","0","0","1","0","0","0","1","0","0","0","0","0","0","0","0","0","1","0","1","0","0","
0","0","0","0","0","0","0","0","0","0","0","0","0","0","0","0","0","0","0","0","0","0","0","0","0","
0","0","0","0","0","0","0","0","0","0","0","0","0","0","0","0","0","28","3","0","NID","tested_neg
ative"

"48","137","70","32","104","0","1","0","1","1","1","1","1","1","0","0","1","0","1","0","0","0","1
","0","1","1","0","0","0","0","0","0","1","1","1","0","0","0","1","0","0","1","0","1","0","1","0","0

","0","0","1","1","1","0","1","0","0","0","0","0","0","0","0","0","0","0","0","0","0","0","0","0
","0","0","0","0","0","0","0","0","0","0","1","0","0","0","0","0","0","14","3","0","NID","tested_p
ositive"

"50","90","80","20","48","0","0","1","0","1","1","0","0","1","0","1","0","1","1","1","1","0","1","
1","0","0","0","0","1","1","0","0","0","1","1","0","0","0","0","0","0","0","1","0","1","1","1","0","
0","0","0","1","0","0","0","0","0","0","0","0","0","0","0","0","0","1","1","0","0","0","0","0","1","
0","1","0","0","0","1","0","0","0","0","1","0","0","0","0","0","0","7","4","0","IND","tested_nega
tive"

"57","135","90","29","89","0","0","0","0","0","0","0","0","0","0","1","0","0","0","0","0","1","0",
"0","0","0","0","0","0","0","0","0","0","0","1","0","0","0","0","1","1","0","0","0","0","0","0","0",
"0","0","0","1","0","1","0","0","0","0","0","0","0","0","0","0","0","0","0","0","0","0","0","0","0",
"1","1","0","0","0","0","0","0","0","0","0","0","0","0","0","0","0","70","2","0","NID","tested_po
sitive"

"85","130","75","18.5","60","0","1","1","1","1","0","0","1","0","0","0","0","1","0","0","0","0","0
","0","0","0","0","0","0","0","0","0","1","0","1","0","0","0","0","0","0","0","0","0","0","1","1","1","0
","0","0","0","0","0","0","0","0","0","1","0","0","0","0","0","0","0","0","0","0","1","1","0","0","0
","0","0","0","0","0","0","0","0","0","1","0","0","0","0","0","0","0","84","2","0","NID","tested_p
ositive"

"55","70","80","24","61","1","1","1","1","1","1","1","1","1","1","1","1","1","1","0","1","1","1","
1","1","1","1","1","1","1","1","1","1","1","1","1","1","0","0","1","1","1","1","1","1","1","1","1","
1","0","0","0","1","1","1","1","0","0","0","0","0","0","0","1","0","0","0","0","1","0","0","0","0","
1","1","1","1","0","1","0","0","0","1","0","0","0","0","0","0","0","90","1","0","NID","tested_neg
ative"

"50","88","80","22","57","1","0","1","0","0","0","0","1","1","0","1","0","0","1","1","1","1","1","
1","1","0","1","0","0","0","0","0","0","1","1","0","0","0","0","1","1","0","1","1","1","1","0","0","
0","0","0","0","0","1","0","1","0","0","0","0","0","0","0","0","0","0","0","0","1","0","0","0","0","
1","1","0","0","0","1","0","0","0","0","1","0","1","0","0","0","0","28","3","0","NID","tested_neg
ative"

"60","129","87","21","58","0","1","1","1","1","1","0","1","0","0","0","0","1","1","1","1","1","1",
"1","1","0","0","0","0","0","0","0","1","1","1","0","0","0","0","0","1","0","1","1","1","1","0","0",
"0","0","0","1","1","1","0","0","0","0","1","0","0","0","0","1","0","0","1","0","1","0","0","0","0",
"1","1","0","0","0","1","0","0","0","0","1","0","0","0","0","0","0","70","4","0","NID","tested_po
sitive"

"53","131","75","19","51","1","1","1","0","1","1","0","1","1","1","0","0","1","1","1","1","0","0",

"1","1","0","0","0","0","0","0","0","1","1","1","0","0","0","0","0","0","0","0","1","1","1","1","0",
"0","0","0","0","0","1","0","0","0","0","0","0","0","0","0","0","0","0","0","0","0","0","0","0","1",
"0","0","1","0","0","0","0","0","0","1","0","0","0","0","0","0","0","14","4","0","NID","tested_po
sitive"

"55","146","80","19.5","58","0","0","1","0","0","0","0","1","0","0","0","0","0","1","1","0","1","1
","1","0","0","0","0","0","0","0","0","0","1","1","0","0","0","0","0","0","0","0","0","1","1","0","0
","0","0","0","0","0","0","0","0","0","0","0","0","0","0","0","0","0","0","0","0","0","0","0","0","1
","0","0","1","0","0","0","0","0","0","0","1","0","0","0","0","0","0","70","2","0","NID","tested_p
ositive"

"57","151","90","19","52","0","0","0","0","0","0","0","0","0","0","0","0","0","0","0","0","0","0",
"0","0","0","0","0","0","0","0","0","0","0","0","0","0","0","0","0","0","0","0","0","1","0","0","0",
"0","0","0","1","0","0","0","0","0","0","0","0","0","0","0","0","0","0","0","0","0","0","0","0","0",
"0","0","0","0","0","0","0","0","0","0","0","0","0","0","0","0","0","42","2","0","NID","tested_po
sitive"

"47","136","80","31","92","1","1","1","0","1","1","0","1","0","0","1","0","1","1","1","1","1","1",
"0","1","0","0","0","0","0","0","0","1","1","1","0","1","0","1","1","0","0","0","0","0","0","1","0",
"0","1","1","1","1","0","0","0","0","0","0","0","0","0","0","0","0","0","0","1","1","1","1","0","0",
"1","1","1","0","0","0","0","0","0","0","1","1","0","0","0","0","1","28","1","0","IND","tested_po
sitive"

"46","68","70","19","58","0","0","1","0","1","0","0","0","1","0","1","0","1","1","0","0","1","1","
0","0","0","0","0","0","0","0","0","0","1","1","0","0","0","0","1","0","0","0","0","1","1","1","0","
0","0","0","0","0","0","0","0","0","0","0","0","0","0","0","1","0","0","0","0","0","0","0","0","0","
0","1","0","0","0","1","0","0","0","0","0","0","0","0","0","0","0","70","3","0","NID","tested_neg
ative"

"40","70","80","18.5","58","0","0","1","0","1","0","0","0","0","0","1","0","1","1","0","1","0","1"
,"1","0","0","0","0","1","1","0","0","0","1","0","0","0","0","0","1","0","0","0","0","1","1","1","0"
,"1","0","0","0","0","0","0","0","0","0","0","0","0","0","0","0","0","0","0","0","0","1","0","0","0"
,"0","1","1","0","1","0","0","0","0","0","1","0","0","0","0","0","0","28","2","0","NID","tested_ne
gative"

"38","90","80","21.5","60","0","0","1","0","1","0","0","0","0","0","1","0","1","0","1","1","0","1"
,"1","0","0","0","0","0","0","0","0","0","1","0","0","1","0","0","1","0","0","0","0","1","1","0","0"
,"0","0","1","0","0","0","0","0","0","0","0","0","0","0","0","0","0","0","0","1","1","0","0","0","0"
,"0","1","1","0","0","0","0","0","0","0","0","0","0","0","0","0","0","90","1","0","NID","tested_ne
gative"

"36","140","90","18","60","1","0","1","1","1","1","0","1","1","1","0","1","1","1","1","1","1","1",
"1","1","0","0","0","0","0","1","0","1","1","1","1","1","0","1","0","1","1","1","1","1","1","1","0",
"0","1","1","1","1","1","1","1","0","1","0","0","0","0","0","0","0","0","1","1","1","0","0","0",
"1","1","1","0","1","1","0","0","0","0","1","0","0","0","0","0","0","7","6","0","NID","tested_posi
tive"

"52","138","70","18","48","0","0","1","0","1","0","0","1","0","0","1","0","1","1","1","1","1","0",
"1","0","0","0","0","0","0","0","0","1","1","0","0","0","0","0","1","0","0","1","0","1","1","1","0",
"0","0","0","0","0","0","0","0","0","1","0","0","0","0","0","0","0","0","0","0","1","0","0","0","0",
"1","1","0","0","0","0","0","0","0","1","1","0","0","0","0","0","0","35","2","0","NID","tested_po
sitive"

"60","140","80","21","73","0","1","0","0","1","0","0","1","1","0","1","1","1","1","0","1","0","1",
"1","0","0","0","0","0","0","0","0","1","0","1","0","1","0","0","0","0","0","0","1","0","1","0","0",
"0","0","0","1","0","0","0","0","0","0","0","0","0","1","0","0","0","0","1","1","0","1","0","0","1",
"0","1","0","0","1","0","0","0","0","1","0","0","0","0","0","0","0","7","3","0","NID","tested_posi
tive"

"70","88","70","18","49","0","1","1","0","1","0","0","1","0","0","0","0","1","0","1","0","1","1","
0","0","0","0","0","0","0","0","0","1","1","1","0","0","0","0","0","0","0","0","0","0","1","0","0","
0","0","0","0","0","0","0","0","0","0","1","0","0","1","0","0","0","0","0","0","0","0","0","0","0","
1","0","0","0","0","0","0","0","0","0","0","0","0","0","0","0","0","70","2","0","NID","tested_neg
ative"

"50","139","75","19.5","65","0","1","1","0","1","1","0","1","0","1","1","1","1","1","1","1","0","0
","1","0","0","0","0","0","1","0","0","1","1","1","1","0","0","0","1","0","0","1","1","1","1","0","1
","0","1","0","0","0","0","0","0","0","0","1","0","0","0","0","1","0","0","0","0","1","0","0","0","0
","1","1","0","0","0","1","0","0","0","0","0","0","0","0","0","0","0","56","2","0","NID","tested_p
ositive"

"75","160","90","26","72","0","1","0","0","1","0","0","1","0","0","1","0","1","0","1","1","1","0",
"0","0","0","0","0","0","0","0","0","1","1","1","0","0","0","0","1","0","0","0","1","0","1","0","0",
"0","0","0","0","0","0","0","0","0","0","1","0","0","0","0","1","0","0","1","0","0","0","0","0","0",
"1","0","0","0","0","0","0","0","0","1","0","0","0","0","0","0","0","28","2","0","NID","tested_po
sitive"

"75","145","80","21","63","0","0","0","0","0","0","0","0","0","0","1","0","0","0","0","0","0","0",
"0","0","0","0","0","0","0","0","0","0","0","1","0","0","0","0","0","0","0","0","0","0","0","0","0",
"0","0","0","1","0","0","0","0","0","0","0","0","0","0","0","0","0","0","0","0","0","0","0","0","0",
"0","0","0","0","0","0","0","0","0","0","0","0","0","0","0","0","0","21","5","0","NID","tested_po

sitive"

"57","170","87","30","94","0","1","0","0","1","0","0","1","0","0","1","0","1","0","1","0","1","1",
"0","0","0","0","0","0","0","0","0","1","0","1","0","0","0","0","1","0","0","0","0","0","0","0","0",
"0","0","0","1","0","0","0","0","0","0","0","0","0","0","0","0","0","0","0","0","0","0","0","0","0",
"0","0","0","0","0","1","0","0","0","0","0","0","0","0","0","0","0","28","4","3","NID","tested_po
sitive"

"53","153","70","19","58","1","1","1","0","1","0","0","1","0","0","1","0","1","1","1","1","1","0",
"0","0","0","0","0","0","0","0","0","1","1","0","0","0","0","0","1","0","0","0","1","1","1","0","0",
"0","0","0","0","0","0","0","0","0","0","0","0","0","0","0","0","0","0","0","0","0","0","0","0","0",
"1","1","1","0","0","1","0","0","0","0","1","0","0","0","0","0","0","14","8","0","NID","tested_po
sitive"

"65","98","84","20","56","1","1","1","0","1","0","0","1","0","0","0","1","1","1","1","1","1","1","
1","0","0","1","0","1","1","1","1","1","1","1","0","0","0","1","1","0","0","0","0","1","1","0","0","
0","0","1","1","1","1","0","0","0","0","1","0","0","0","0","1","0","0","0","1","1","1","0","0","1","
1","0","1","1","0","0","0","0","1","0","1","0","0","0","0","0","0","28","3","0","NID","tested_neg
ative"

"58","94","70","22","62","1","1","1","1","1","1","0","0","0","0","1","1","1","0","1","1","0","1","
1","1","1","0","0","1","1","0","0","1","1","0","0","0","0","0","1","0","0","0","0","1","1","0","0","
0","0","0","0","0","0","0","0","0","0","1","0","0","0","0","1","0","0","0","0","0","0","0","0","0","
1","0","1","0","0","1","0","0","0","0","0","0","0","0","0","0","0","28","3","0","NID","tested_neg
ative"

"42","170","80","28","80","0","0","1","0","0","0","0","0","0","0","0","0","0","0","0","0","0","0",
"0","0","0","0","0","0","0","0","0","0","0","0","0","0","0","0","0","0","0","0","1","0","0","0","0",
"0","0","0","0","0","0","0","0","0","0","0","0","0","0","0","0","0","0","0","0","0","0","0","0","0",
"0","0","0","0","0","0","0","0","0","0","0","0","0","0","0","0","0","70","2","0","NID","tested_po
sitive"

"68","152","70","20.5","60","0","0","0","0","0","0","0","1","0","0","0","0","0","0","0","0","0","0
","0","0","0","0","0","0","0","0","0","0","0","1","0","0","0","0","0","0","0","0","0","0","1","0","0
","0","0","0","0","0","0","0","0","0","0","0","0","0","0","0","0","0","0","0","0","0","0","0","0","0
","0","0","0","0","0","0","0","0","0","0","0","0","0","0","0","0","0","28","6","0","NID","tested_p
ositive"

"40","141","80","19","52","1","1","1","0","0","0","0","1","1","1","1","1","0","1","1","0","1","1",
"1","1","1","0","0","1","1","0","0","1","1","1","0","1","0","0","1","0","1","1","1","1","1","1","0",
"0","1","0","0","0","1","0","0","1","0","0","0","0","0","0","0","0","0","0","0","1","0","0","0","0",

"1","1","0","0","0","1","0","0","0","0","1","0","0","0","0","0","0","7","5","0","NID","tested_posi
tive"

"63","140","86","25","79","1","1","1","1","1","0","1","1","1","0","1","0","1","1","1","1","1","1",
"0","0","0","0","0","0","0","0","0","1","1","1","0","0","0","0","0","1","0","0","0","1","1","0","0",
"0","0","0","1","0","1","0","0","0","0","1","0","0","0","0","0","0","0","0","0","0","0","0","0","1",
"1","1","1","0","0","1","0","0","0","0","1","0","0","0","0","0","0","70","1","0","NID","tested_po
sitive"

"58","95","90","20","65","1","1","1","0","0","0","0","0","0","0","1","0","0","1","1","1","0","1","
0","0","1","0","0","1","1","0","0","1","1","1","0","0","0","0","0","1","1","1","1","1","1","0","0","
0","0","1","0","0","1","1","0","0","0","0","0","0","0","0","0","0","0","0","0","0","0","0","0","0","
0","1","0","0","0","1","0","0","0","0","1","0","0","0","0","0","0","90","2","0","NID","tested_neg
ative"

"45","144","85","19","65","1","1","1","0","1","0","1","0","0","0","1","0","1","1","0","0","0","1",
"0","0","0","0","0","0","0","0","0","1","1","0","0","0","0","0","0","0","0","0","1","1","1","0","0",
"0","0","0","1","0","0","0","0","0","0","0","0","0","0","0","0","0","0","0","0","0","0","0","0","0",
"0","0","0","0","0","1","0","0","0","0","1","0","0","0","0","0","0","56","2","0","NID","tested_po
sitive"

"63","150","80","22","72","0","0","1","0","1","0","0","1","1","0","0","0","1","1","1","1","0","0",
"0","0","0","0","0","0","0","0","0","0","1","1","0","0","0","0","0","1","0","0","0","1","1","0","0",
"0","0","0","0","0","1","0","0","0","0","0","0","0","0","0","0","0","0","0","0","0","0","0","0","0",
"0","0","0","0","0","0","0","0","0","0","1","0","0","0","0","0","0","14","8","0","NID","tested_po
sitive"

"80","140","70","18","58","1","1","1","0","1","0","0","1","0","0","1","0","1","1","1","1","0","1",
"0","0","0","0","0","1","1","0","0","1","1","1","0","0","0","0","0","1","0","0","1","1","1","0","0",
"0","0","0","0","0","1","0","0","0","0","1","1","1","0","0","0","0","0","0","0","1","0","0","0","1",
"0","0","0","0","0","1","0","0","0","0","1","0","0","0","0","1","0","28","3","0","IND","tested_po
sitive"

"53","136","80","25","78","0","1","1","1","1","1","1","1","0","0","0","0","1","1","1","0","0","0",
"1","0","0","0","0","0","0","0","0","0","0","0","0","0","0","0","0","0","0","0","0","0","0","0","0",
"0","0","0","0","0","0","0","0","0","0","1","0","0","1","1","0","0","0","0","0","0","0","0","0","0",
"0","0","0","0","0","0","0","0","0","0","0","0","0","0","0","0","0","77","2","1","NID","tested_po
sitive"

"55","133","75","20","65","0","0","1","0","0","0","0","0","0","0","0","0","0","0","0","0","0","0",
"0","0","0","0","0","0","0","0","0","0","0","1","0","0","0","0","0","0","0","0","0","0","1","0","0",

"0","0","0","0","0","0","0","0","0","0","0","0","0","0","0","0","0","0","0","0","0","0","0","0","0",
"0","0","0","0","0","1","0","0","0","0","1","0","0","0","0","0","0","70","3","0","NID","tested_po
sitive"

"59","78","70","22.5","80","1","1","1","1","1","0","0","1","1","0","1","0","1","1","1","1","1","1"
,"0","0","0","0","0","0","0","0","0","1","1","1","0","0","0","0","0","1","0","1","1","1","0","0","0"
,"0","0","0","0","0","1","0","0","0","0","0","0","0","0","0","0","0","0","0","0","0","0","0","0","0"
,"0","0","1","0","0","0","0","0","0","0","1","0","0","0","0","0","0","56","2","0","NID","tested_ne
gative"

"77","124","88","26","88","0","0","0","0","1","0","0","0","0","0","1","0","1","1","0","0","0","1",
"1","0","0","0","0","1","1","0","0","0","1","1","0","1","0","0","1","0","0","0","0","0","1","0","0",
"0","0","0","1","0","0","0","0","0","0","0","0","0","0","0","0","0","0","0","0","0","0","0","0","0",
"0","0","0","0","0","0","0","0","0","0","1","0","0","0","0","0","0","42","3","0","NID","tested_po
sitive"

"50","101","90","19","60","1","1","1","0","1","0","0","1","0","1","1","0","1","1","1","0","0","0",
"0","0","0","0","0","0","0","0","0","1","1","0","0","0","0","0","0","0","0","0","0","1","1","0","0",
"0","0","0","0","0","0","0","0","0","0","0","0","0","0","0","0","0","0","0","0","1","1","0","0","0",
"0","0","0","0","0","1","0","0","0","0","1","0","0","0","0","0","0","70","2","0","NID","tested_ne
gative"

"78","89","60","24","65","0","0","1","0","0","0","0","0","1","0","0","0","1","1","0","0","1","0","
1","0","0","0","0","0","0","0","0","0","1","1","0","0","0","1","0","0","0","0","0","1","1","0","0","
0","0","0","1","0","0","0","0","0","0","0","0","0","0","0","0","0","0","0","0","0","0","0","0","1","
1","0","1","0","0","1","0","0","0","0","0","0","0","0","0","0","0","28","3","0","NID","tested_neg
ative"

"75","130","80","22","72","1","1","1","1","1","0","0","0","0","0","1","0","1","1","1","0","1","0",
"1","0","0","0","0","0","0","0","0","1","1","1","0","0","0","0","0","0","0","0","0","1","1","0","0",
"0","0","0","1","0","0","0","0","0","0","0","0","0","0","0","0","0","0","0","0","0","0","0","0","1",
"0","0","1","0","0","1","0","0","0","0","0","0","0","0","0","0","0","82","2","0","NID","tested_po
sitive"

"65","97","70","21","61","1","1","1","0","1","0","0","0","0","0","0","0","1","0","1","1","0","0","
0","0","0","0","0","0","0","0","0","1","0","0","0","0","0","0","0","0","0","0","0","1","0","0","0","
0","0","0","0","0","0","0","0","0","0","0","0","0","0","0","0","0","0","0","0","0","0","0","0","0","
0","0","0","0","0","1","0","0","0","0","1","0","0","0","0","0","0","77","2","0","NID","tested_neg
ative"

"50","144","90","27","85","1","1","1","1","1","0","0","1","1","1","1","1","1","1","1","1","1","0",

"0","1","0","0","0","1","1","0","0","1","1","1","0","0","0","0","0","1","1","1","1","1","1","0","0",
"0","0","0","1","0","1","1","0","0","0","1","0","0","0","0","0","0","0","0","0","1","0","0","0","0",
"1","1","1","0","0","1","0","0","0","0","1","0","0","0","0","0","0","56","2","0","NID","tested_po
sitive"

"67","124","86","23","70","0","0","0","0","0","0","0","0","0","0","0","0","0","0","0","0","0","1",
"0","0","0","0","0","0","0","0","0","0","0","0","0","0","0","0","0","0","0","1","0","0","0","0","0",
"0","0","0","1","0","0","0","0","0","0","0","0","0","0","0","0","0","0","0","0","0","0","0","0","0",
"0","0","0","0","0","0","0","0","0","0","0","0","0","0","0","0","0","56","1","0","NID","tested_po
sitive"

"70","156","90","28","85","1","1","1","1","1","1","0","0","1","0","1","0","1","1","1","1","1","1",
"1","1","0","1","0","1","1","0","1","1","1","1","0","1","0","0","0","1","0","0","0","0","0","0","0",
"0","0","0","1","0","1","0","0","0","0","1","1","0","0","0","0","0","0","0","0","1","1","1","0","0",
"1","1","1","0","1","1","0","0","0","1","1","0","0","0","0","0","0","90","1","1","NID","tested_po
sitive"

"53","131","70","18.5","55","1","0","0","0","0","0","1","1","0","0","0","0","0","1","0","1","0","0
","0","0","0","1","0","0","0","0","0","0","1","0","0","0","0","0","0","0","0","0","0","0","0","0","0
","0","0","0","0","0","0","0","0","0","0","0","0","0","0","0","0","0","0","0","0","0","0","0","0","0
","0","0","0","0","0","0","0","0","0","0","1","0","0","0","0","0","0","28","2","0","NID","tested_p
ositive"

"56","88","90","20","70","0","0","0","0","0","0","0","0","0","0","1","0","0","0","0","0","0","0","
0","0","0","0","0","0","0","0","0","0","0","0","0","0","0","0","0","1","0","0","0","0","0","0","0","
0","0","0","1","0","1","0","0","0","0","0","0","0","0","0","0","0","0","0","0","0","0","0","0","0","
0","1","0","0","0","0","0","0","0","0","1","0","0","0","0","0","0","28","6","0","NID","tested_neg
ative"

"52","105","80","29","85","0","0","1","0","1","0","0","0","1","1","0","0","0","1","0","0","0","0",
"1","0","0","0","0","0","0","0","0","0","1","1","0","1","0","0","0","0","0","0","1","1","0","0",
"0","0","0","1","0","0","0","0","0","0","0","0","0","0","0","0","0","0","0","0","0","0","0","0","0",
"0","1","0","0","0","1","0","0","0","0","1","0","0","0","0","0","0","7","4","1","NID","tested_posi
tive"

"55","70","70","23.5","78","0","0","1","0","0","0","0","0","0","0","1","0","0","0","0","0","0","0"
,"0","0","0","0","0","1","1","0","0","0","0","0","0","0","0","0","1","0","0","0","0","1","1","0","0"
,"0","0","0","0","0","0","0","0","0","1","0","0","0","0","0","0","0","0","0","0","0","0","0","0","0"
,"0","1","0","0","1","1","0","0","0","1","0","0","0","0","0","0","0","14","1","0","NID","tested_ne
gative"

"38","121","80","21","62","1","0","1","0","0","0","0","0","0","0","0","0","0","1","0","0","0","0",
"0","0","0","1","0","0","0","0","0","0","1","0","0","0","0","0","0","1","0","0","1","1","1","0","0",
"0","0","0","0","0","1","0","0","0","0","0","0","0","0","0","0","0","0","0","0","1","1","0","0","0",
"0","0","0","0","1","1","0","0","1","0","0","0","0","0","0","0","0","7","5","0","NID","tested_posi
tive"

"59","133","75","20","49","1","1","1","0","1","0","0","1","1","0","1","1","1","1","1","0","1","1",
"1","1","0","0","0","0","1","1","1","1","1","0","1","0","0","0","1","1","0","0","1","1","1","1","1",
"1","0","0","0","1","1","0","1","0","1","1","0","0","1","0","0","0","0","1","0","0","0","0","0","0",
"1","1","1","1","1","1","0","0","0","1","1","0","0","0","0","0","0","7","2","0","NID","tested_posi
tive"

"70","90","90","29","88","1","1","1","1","0","0","1","0","0","0","1","0","0","0","1","0","0","1","
0","0","1","1","0","1","0","0","0","1","1","0","0","0","0","0","1","1","1","0","1","1","1","1","0","
0","0","0","1","1","1","0","0","0","1","0","0","0","0","0","0","0","0","0","0","0","0","0","0","0","
0","0","0","0","1","1","0","0","1","1","0","0","0","0","0","0","0","360","0","0","NID","tested_ne
gative"

"39","85","70","18","58","0","1","0","0","0","0","0","0","0","0","0","0","0","0","0","1","0","0","
1","0","0","0","0","0","0","0","0","0","1","0","0","0","0","0","0","1","0","0","1","0","0","1","0","
0","0","0","0","1","1","0","0","0","1","0","0","0","0","0","0","0","0","0","0","0","1","0","0","0","
0","1","1","0","1","0","0","0","0","1","0","0","0","0","0","0","0","30","2","0","NID","tested_neg
ative"

"50","71","90","21","68","1","1","0","1","1","1","0","0","0","0","1","1","1","1","0","1","0","1","
0","1","1","1","0","0","1","1","0","1","1","1","1","0","0","0","1","0","1","1","1","0","1","1","1","
1","0","0","1","1","0","1","0","0","1","1","0","0","0","0","0","0","0","0","0","0","0","0","0","0","
0","1","0","1","1","0","0","0","1","1","0","0","0","0","0","0","0","60","2","0","NID","tested_neg
ative"

"65","141","83","26","76","0","1","1","0","1","0","0","0","0","0","0","0","1","1","1","1","1","0",
"0","0","0","0","0","0","0","0","0","1","1","1","0","0","0","0","0","0","0","0","0","1","1","1","0",
"0","0","0","1","0","0","0","0","0","0","1","0","0","0","0","0","0","0","0","0","0","0","0","0","0",
"1","1","0","0","0","1","0","0","0","0","1","0","0","0","0","0","0","21","2","0","NID","tested_po
sitive"

"50","137","68","22","66","1","0","1","0","0","0","0","0","0","0","1","0","0","1","0","0","1","0",
"0","0","0","0","0","0","0","0","0","0","1","1","0","0","0","0","0","0","0","0","1","1","0","0","0",
"0","0","0","0","0","0","0","0","0","0","0","0","0","0","0","0","0","0","0","0","1","0","0","0","0",
"0","1","1","0","0","1","0","0","0","0","1","0","0","0","0","0","0","63","3","0","NID","tested_po

sitive"

"33","77","70","27","80","1","1","0","0","0","0","0","0","1","0","1","0","0","1","1","0","0","0","
0","0","0","0","0","0","0","0","0","1","1","0","0","0","0","0","1","0","0","0","1","0","1","0","0","
1","0","0","0","0","0","0","0","0","0","0","1","1","0","0","0","0","0","0","0","1","1","0","0","0","
1","1","0","0","0","0","0","0","0","0","1","0","0","0","0","0","0","42","2","0","NID","tested_neg
ative"

"45","200","82","18","65","0","0","1","0","0","0","0","0","1","0","0","0","0","0","0","0","1","0",
"0","0","0","0","0","0","1","0","0","0","1","0","0","0","0","0","0","0","0","0","1","1","0","0","0",
"0","0","0","1","0","0","0","0","0","0","0","0","0","0","0","0","0","0","0","0","0","0","0","0","0",
"1","0","0","0","0","1","0","0","0","0","1","0","0","0","0","0","0","42","4","0","NID","tested_po
sitive"

"56","174","88","20","65","1","1","1","0","0","1","0","1","0","1","0","1","0","1","1","1","1","0",
"0","1","0","0","0","0","0","0","0","1","1","0","0","0","0","0","0","1","0","1","0","0","1","1","0",
"0","0","0","0","0","1","0","0","0","0","0","0","0","0","0","0","0","0","0","0","0","0","0","0","0",
"1","1","1","1","1","1","0","0","1","1","1","0","0","0","0","0","0","28","4","0","NID","tested_po
sitive"

"40","147","80","30","98","0","1","0","0","0","0","0","0","0","0","1","0","0","0","0","1","1","1",
"0","0","0","0","0","0","0","0","0","1","0","0","0","0","0","0","1","0","0","0","0","0","0","1","0",
"0","0","0","1","1","0","0","0","0","0","0","0","0","0","0","0","0","0","0","0","0","0","0","0","0",
"1","1","0","0","1","0","0","0","1","0","0","0","0","0","0","0","0","56","3","0","NID","tested_po
sitive"

"49","89","70","20","59","0","1","1","0","0","0","0","0","0","0","0","0","0","1","0","0","0","
1","0","0","0","0","0","1","0","0","1","0","1","0","0","0","1","0","1","0","0","1","1","1","0","0","
0","0","0","0","0","1","0","0","0","0","0","0","0","0","0","0","0","0","0","1","0","0","0","0","0","
0","0","1","0","0","0","0","0","0","1","0","0","0","0","0","0","0","35","6","0","NID","tested_neg
ative"

"65","130","60","18.5","52","1","1","1","1","1","1","0","1","0","0","1","1","1","1","1","1","1","0
","0","0","0","0","0","1","1","0","0","1","1","1","0","0","0","0","1","1","0","0","0","1","1","0","0
","0","0","0","0","1","1","0","0","0","1","0","0","0","0","0","0","0","0","0","0","0","1","0","0","1
","1","0","1","0","0","1","0","0","0","1","1","0","0","0","0","0","0","14","4","0","NID","tested_p
ositive"

"80","153","86","17","48","0","0","0","0","0","0","0","0","0","0","0","1","0","0","0","0","0",
"0","0","0","0","0","0","0","0","0","0","0","0","0","0","0","0","0","0","0","0","0","0","0","0","0",
"0","0","1","1","0","0","0","0","0","0","0","0","0","0","0","1","0","0","0","0","0","0","0","0","0",

"0","0","0","0","0","0","0","0","0","0","1","0","0","0","0","0","0","14","5","0","NID","tested_po
sitive"

"57","128","98","25","75","1","1","1","0","1","0","0","0","1","1","1","1","1","1","1","1","1","0",
"1","1","0","0","0","1","1","0","0","1","1","0","0","0","0","0","1","1","0","0","0","1","0","1","0",
"0","0","0","1","1","1","0","0","0","1","0","0","0","0","0","0","0","0","0","0","0","1","0","0","0",
"1","1","0","0","1","1","0","0","0","0","1","0","0","0","0","0","0","28","3","0","NID","tested_po
sitive"

"55","140","70","25.5","75","0","0","0","0","0","0","0","1","1","0","0","0","1","0","0","0","1","0
","0","0","0","0","0","0","0","0","0","0","1","0","0","0","0","0","0","0","0","0","0","1","0","0
","0","0","0","0","0","0","0","0","0","0","0","0","0","0","0","0","0","0","0","0","0","1","0","0","0
","0","0","0","0","0","0","0","0","0","0","1","0","0","0","0","0","0","14","3","0","NID","tested_p
ositive"

"50","137","70","26","80","0","0","0","0","1","0","0","0","0","0","0","0","1","1","0","0","0","0",
"0","0","0","0","0","0","0","0","0","0","0","0","0","0","0","0","0","0","0","0","0","0","0","0","0",
"0","0","0","0","0","0","0","0","0","0","0","0","0","0","0","0","0","0","0","0","0","0","0","0","0",
"0","0","0","0","0","0","0","0","0","0","0","0","0","0","0","0","0","42","2","0","NID","tested_po
sitive"

"60","90","68","19.5","65","0","1","0","0","0","0","0","1","0","0","0","1","0","1","1","0","0","0"
,"1","0","0","0","0","0","0","0","0","1","1","0","0","1","0","0","0","0","0","0","0","0","1","0"
,"0","0","0","0","0","0","0","0","0","0","0","0","0","0","0","0","0","0","0","0","0","0","0","0","0"
,"0","0","0","0","0","0","0","0","0","1","0","0","0","0","0","0","0","7","1","0","NID","tested_neg
ative"

"65","158","70","22","76","0","1","1","0","1","0","0","0","0","0","0","1","1","0","1","0","0",
"0","0","0","0","1","1","1","0","0","1","1","1","0","0","0","0","1","1","0","0","0","0","1","1","0",
"0","0","0","0","0","1","0","0","0","1","1","0","1","0","0","0","0","0","0","0","0","1","0","0","0",
"0","0","1","1","1","1","1","0","0","1","0","0","0","0","0","0","0","7","12","0","NID","tested_po
sitive"

"30","203","80","19","75","0","1","1","1","1","0","0","1","0","0","1","0","1","1","0","0","1","1",
"0","0","1","0","0","1","1","0","0","1","1","1","0","0","0","0","1","0","1","0","0","0","1","1","0",
"1","0","0","0","0","0","1","0","0","1","1","0","0","0","0","0","0","0","0","0","0","1","0","0","0",
"1","1","0","1","1","1","1","0","0","1","1","0","0","0","0","0","0","49","4","0","NID","tested_po
sitive"

"45","91","70","19","50","0","0","1","0","1","1","0","0","0","1","0","0","1","0","0","0","0","0","
0","0","0","0","0","0","0","0","0","0","0","0","0","0","0","0","0","0","0","0","0","0","0","0","0","

0","0","0","0","0","0","0","0","0","0","1","0","0","0","0","0","0","0","0","0","0","0","0","0","0","
0","0","0","0","0","0","0","0","0","0","0","0","0","0","0","0","0","56","2","0","NID","tested_neg
ative"

"80","66","90","18","35","1","1","1","1","1","1","1","0","0","0","0","0","0","1","1","1","0","0","
0","0","0","0","0","0","0","0","0","1","1","0","0","0","0","0","1","1","0","1","1","0","1","1","0","
0","0","0","1","0","0","0","0","1","0","1","0","0","0","0","0","1","0","0","0","0","1","0","0","0","
0","0","0","0","1","0","0","0","0","0","0","0","0","0","0","0","0","60","1","0","NID","tested_neg
ative"

"48","86","80","19","55","1","1","0","0","0","0","0","0","0","0","0","0","0","1","0","0","0","0","
0","0","0","0","0","0","0","0","0","1","1","0","0","0","0","0","0","1","1","1","0","0","1","1","0","
0","0","0","0","0","0","0","1","1","1","0","0","0","0","0","0","0","0","1","0","0","1","0","0","0","
0","0","0","0","1","0","0","0","0","0","0","0","0","0","0","0","0","90","2","2","NID","tested_neg
ative"

"25","162","80","18.5","48","1","1","0","0","0","0","0","0","0","1","0","1","1","0","0","0","0
","0","0","0","0","0","0","0","0","0","1","1","1","0","0","0","0","0","0","0","0","0","0","1","1","0
","0","0","0","0","0","1","0","0","0","1","0","0","0","1","0","0","1","0","0","0","0","0","0","0","0
","0","0","0","0","0","0","0","0","0","1","0","0","0","0","0","0","0","14","6","0","NID","tested_p
ositive"

"40","171","70","19.5","60","1","1","0","1","1","0","1","0","0","0","0","0","0","1","1","1","0","0
","0","0","0","0","0","0","0","0","0","1","1","1","0","0","0","0","0","1","0","1","0","0","1","1","0
","0","0","0","0","0","1","0","0","0","1","0","0","1","0","0","0","0","0","0","0","0","1","0","0","0
","0","0","0","0","0","0","0","0","0","1","0","1","0","0","0","0","1","7","6","0","NID","tested_po
sitive"

"40","155","60","23","89","1","1","1","0","0","0","0","0","0","1","0","0","1","0","0","0","0",
"0","0","0","0","0","0","0","0","0","1","1","0","0","0","0","0","0","0","0","0","0","0","1","0","0",
"0","0","0","0","0","0","0","0","0","0","0","0","0","0","0","0","0","0","1","0","0","0","0","0","0",
"0","0","0","0","0","1","0","0","0","0","0","0","0","0","0","0","0","80","6","6","NID","tested_po
sitive"

"58","149","70","22.5","73","1","1","0","0","0","0","0","0","0","0","0","0","0","1","1","1","1
","0","0","0","1","0","0","0","0","0","1","1","0","0","0","0","0","0","1","0","0","0","0","0","0","0
","0","0","0","0","0","1","0","0","0","0","0","0","0","0","0","0","0","0","0","0","0","0","0","0","0
","0","0","0","0","0","0","0","0","0","0","0","0","0","0","0","0","0","30","3","0","NID","tested_p
ositive"

"68","71","80","24","70","0","1","0","0","1","0","0","0","0","0","1","0","0","0","1","0","0","0","

0","0","0","0","0","0","0","0","0","1","0","0","0","0","0","0","0","0","0","0","0","0","0","0","0","
0","0","0","0","0","0","0","0","0","0","0","0","1","0","0","0","0","0","0","0","0","0","0","0","0","
0","0","0","0","0","0","0","0","0","0","0","0","0","0","0","0","0","28","2","0","NID","tested_neg
ative"

"50","211","80","30.5","98","1","1","0","1","1","0","0","0","0","0","1","0","0","0","0","0","0","0
","0","0","0","0","0","0","0","0","0","1","0","0","0","0","0","0","0","0","0","0","0","0","0","0","0
","0","0","0","0","0","0","0","0","0","0","0","0","0","0","0","0","0","0","0","0","0","0","0","0","0
","0","0","0","0","0","0","0","0","0","0","0","1","0","0","0","0","1","14","5","3","GTD","tested_
positive"

"39","181","90","28","93","1","1","1","1","0","0","0","0","0","1","1","1","1","1","1","1","1",
"1","1","1","1","1","1","1","1","0","1","1","0","1","0","0","0","0","1","1","1","0","0","1","1","1",
"1","0","0","1","0","1","1","1","0","1","1","1","1","0","0","1","0","0","1","0","0","0","0","0","0",
"0","0","0","0","1","0","0","0","0","1","0","0","0","0","0","0","0","30","6","1","NID","tested_po
sitive"

"55","65","70","20","98","1","1","1","0","0","0","0","0","0","0","0","0","0","0","0","0","0","0","
0","0","0","0","0","0","0","0","0","1","0","0","0","0","0","0","0","0","0","0","0","1","0","0","0","
0","0","0","0","0","0","0","0","0","0","0","0","0","0","0","0","0","0","0","0","0","0","0","0","0","
0","0","0","0","0","0","0","0","0","0","0","0","0","0","0","0","0","7","6","2","NID","tested_nega
tive"

"37","165","88","19","60","1","0","0","1","0","0","0","0","0","0","0","0","1","0","0","0","1",
"0","0","0","0","0","0","0","0","0","0","1","0","0","0","0","1","0","1","0","1","0","0","0","0","0",
"0","0","0","1","0","1","0","0","0","1","0","0","0","0","0","0","0","0","0","0","1","0","0","0",
"0","0","0","0","1","0","0","0","0","1","0","1","0","0","0","0","1","14","2","0","NID","tested_po
sitive"

"59","78","70","33","86","1","1","1","0","0","0","0","0","0","0","0","0","0","1","0","0","0","0","
0","0","0","0","0","0","0","0","0","1","1","0","0","0","0","0","0","0","0","0","0","0","1","0","0","
0","0","0","0","0","0","0","0","0","0","0","0","0","0","0","0","0","0","0","0","0","0","0","0","0","
0","0","0","0","0","0","0","0","0","1","0","0","0","0","0","0","0","60","3","1","NID","tested_neg
ative"

"73","61","68","19","59","0","1","0","0","0","0","0","0","0","0","0","0","0","0","0","0","0","
0","0","0","0","0","0","0","0","0","1","0","0","0","0","0","0","0","0","0","0","0","0","0","0","0","
0","0","0","0","0","0","0","0","0","0","0","0","0","0","0","0","0","0","0","0","0","0","0","0","0","
0","0","0","0","0","0","0","0","0","0","0","0","0","0","0","0","0","21","1","0","NID","tested_neg
ative"

"60","144","70","27","93","1","1","1","1","1","0","0","0","0","1","1","1","1","1","0","0","0","0",
"0","0","0","0","0","0","0","0","0","1","1","1","0","0","0","0","0","0","1","1","1","1","0","0","0",
"0","0","0","0","0","1","0","0","0","0","0","0","0","0","0","0","0","0","0","0","0","0","0","0","0",
"0","0","0","0","0","0","0","0","0","0","0","1","0","0","0","0","1","30","6","3","GTD","tested_po
sitive"

"50","152","90","25.5","80","1","1","1","1","1","0","0","0","0","0","0","0","1","1","0","0","0","0
","0","0","0","0","0","0","0","0","0","1","1","0","0","0","0","0","0","0","0","0","0","0","0","1","0
","0","0","0","1","0","0","0","0","0","0","0","0","1","0","0","0","0","0","1","0","0","1","0","0","0
","0","0","0","0","1","1","0","0","0","0","1","0","0","0","0","0","0","30","6","1","NID","tested_p
ositive"

"59","166","87","23","80","1","1","1","0","0","0","0","0","0","0","1","1","0","0","0","0","0","0",
"0","0","0","0","0","0","0","0","0","1","0","0","0","0","0","0","0","0","0","0","0","0","0","0","0",
"0","0","0","1","0","0","0","0","0","0","0","0","0","0","0","0","0","0","0","0","0","0","0","0","0",
"0","0","0","0","0","0","0","0","0","0","0","1","0","0","0","0","1","14","2","1","NID","tested_po
sitive"

"75","142","90","20","69","1","1","1","1","1","0","0","0","0","0","1","0","1","1","1","1","0",
"0","0","0","0","0","0","0","0","0","1","1","0","0","0","0","0","0","0","0","0","0","0","1","0","0",
"0","0","0","1","0","0","0","0","0","0","0","0","0","0","0","0","0","0","0","0","0","0","0","0","0",
"0","0","0","0","0","1","0","0","0","0","0","0","0","1","0","0","0","70","5","2","NID","tested_po
sitive"

"31","139","80","18","50","1","1","1","0","0","0","0","0","1","1","1","1","0","0","0","1","0","1",
"0","0","1","0","0","0","0","0","0","1","1","0","0","0","0","0","0","1","1","1","0","1","1","0","0",
"0","0","0","1","0","1","1","1","1","1","0","0","0","0","0","0","0","0","0","0","1","1","0","0","0",
"0","0","0","1","0","1","0","0","0","1","0","1","0","0","0","0","1","7","3","0","NID","tested_posi
tive"

"50","117","60","31","93","1","1","1","0","1","0","0","0","0","0","0","0","0","0","0","0","0","0",
"0","0","0","0","0","0","1","0","0","1","0","0","0","0","0","0","0","1","0","0","0","1","0","0","0",
"1","0","0","0","0","1","0","0","0","0","0","1","0","0","0","0","0","0","1","0","0","0","0","0","0",
"0","0","0","0","0","1","0","0","0","0","0","0","0","0","0","0","0","30","6","0","NID","tested_po
sitive"

"48","122","93","23","83","1","1","1","1","0","0","0","0","0","0","1","1","1","1","1","1","0","0",
"0","0","1","0","0","0","0","0","0","1","1","0","0","0","0","0","0","1","1","0","0","0","1","0","0",
"0","0","0","1","0","1","1","0","0","0","1","1","0","0","0","0","0","0","1","0","0","1","0","0","0",
"0","0","0","0","0","0","0","0","0","0","0","1","0","0","0","0","1","10","7","0","GTD","tested_po

sitive"

"38","118","80","20.5","62","1","1","1","1","1","0","0","1","0","0","1","0","1","1","1","1","1","0
","1","0","0","0","0","0","0","0","0","1","1","0","0","0","0","0","0","1","0","1","0","0","1","0","0
","0","0","0","1","0","1","0","0","1","0","0","0","1","0","0","0","0","1","0","0","0","1","0","1","0
","0","0","0","0","1","0","0","0","0","1","1","1","0","0","0","0","1","7","3","2","NID","tested_po
sitive"

"49","181","87","19.5","83","1","1","1","1","1","0","0","1","0","0","1","0","1","1","1","1","0","1
","0","0","0","0","0","0","0","0","0","1","1","0","0","0","0","0","0","1","0","1","0","0","1","0","0
","0","0","0","1","0","1","0","0","1","0","0","0","1","0","0","0","0","1","0","0","0","0","0","1","0
","0","0","0","0","0","0","0","0","0","0","1","1","0","0","0","0","1","30","2","2","NID","tested_p
ositive"

"45","78","90","18","55","1","1","1","1","0","0","0","0","0","0","1","1","1","1","1","0","0","1","
0","0","1","0","0","0","0","0","1","1","1","0","0","0","0","0","0","1","0","1","1","1","1","1","0","
0","0","0","1","0","1","0","0","0","1","0","0","1","0","0","0","1","0","0","0","0","1","0","0","0","
0","0","0","0","1","1","0","0","0","1","1","0","0","0","0","0","0","14","8","4","NID","tested_neg
ative"

"48","125","80","21","67","0","1","0","1","0","0","0","0","0","0","1","0","1","1","0","1","0","1",
"0","0","0","0","0","0","0","0","0","1","1","0","0","0","0","0","0","0","1","1","0","0","1","1","0",
"0","0","0","1","0","0","1","0","0","1","0","0","0","0","0","0","0","0","0","0","0","1","0","0","0",
"0","0","0","0","1","1","0","0","0","1","0","0","0","0","0","0","0","7","4","3","NID","tested_posi
tive"

"41","119","90","22.5","61","1","1","1","1","0","0","0","0","0","0","1","0","0","1","1","0","0","0
","0","0","0","0","0","0","0","0","0","1","1","0","0","1","0","0","0","1","0","1","0","1","1","1","0
","0","0","0","1","0","1","0","0","1","1","0","0","0","0","0","0","0","0","0","0","0","1","0","0","0
","1","0","1","0","1","1","0","0","0","1","0","0","0","0","0","0","0","60","4","0","NID","tested_p
ositive"

"60","94","80","19.5","63","0","0","0","1","1","0","0","0","0","0","0","0","0","1","0","0","0","1"
,"0","0","0","0","0","0","0","0","0","0","1","0","0","0","0","0","0","1","1","1","0","0","1","1","0"
,"0","0","0","0","0","1","0","1","1","0","0","0","0","0","0","0","0","0","0","0","0","1","0","0","0"
,"0","0","0","0","1","0","0","0","0","1","0","0","0","0","0","0","0","210","0","0","GTD","tested_
negative"

"33","151","90","18","55","0","1","1","1","0","0","0","0","0","0","0","0","1","1","0","0","0","1",
"0","0","0","0","0","0","0","0","0","0","1","1","0","0","0","0","0","1","0","1","0","0","1","1","0",
"0","0","0","1","0","1","0","0","0","1","0","0","0","0","0","0","0","0","0","0","0","1","0","0","0",

"0","0","1","0","1","0","0","0","0","1","0","0","0","0","0","0","0","21","2","2","NID","tested_po
sitive"

"28","133","87","20","63","1","1","1","1","0","0","0","0","0","0","0","0","0","1","0","0","0","0",
"1","0","1","0","0","0","0","0","0","1","1","0","0","0","0","0","0","0","1","0","0","1","1","1","0",
"0","0","0","1","0","0","1","0","0","1","0","0","0","0","0","0","0","0","0","0","0","1","0","0","0",
"0","0","1","0","1","1","0","0","0","1","0","0","0","0","0","0","0","2","4","3","NID","tested_posi
tive"

"45","121","70","19","56","1","1","1","0","0","0","0","0","0","0","0","0","0","1","0","0","0","0",
"0","0","0","0","0","0","0","0","0","1","1","1","0","0","0","0","0","0","0","0","0","0","1","1","0",
"0","0","0","0","0","0","0","0","0","1","0","0","0","0","0","0","0","0","0","0","0","1","0","0","0",
"0","0","0","0","1","0","0","0","0","1","0","0","0","0","0","0","0","60","1","0","NID","tested_po
sitive"

"39","144","90","28","89","1","1","1","1","0","0","0","0","0","0","0","0","1","1","1","0","0",
"0","0","0","0","0","0","0","0","0","1","1","1","0","0","0","0","0","0","0","0","0","1","1","1","0",
"0","0","0","1","0","0","0","0","0","0","0","0","0","0","0","0","0","0","0","0","0","1","0","0","0",
"0","0","0","0","1","0","0","0","0","1","0","0","0","0","0","0","0","30","6","0","NID","tested_po
sitive"

"80","130","100","18","45","1","1","1","1","1","1","1","1","1","0","0","0","0","1","0","0","0","0
","0","0","0","0","0","0","0","0","0","1","1","1","0","0","0","0","0","0","0","0","0","1","1","1","0
","0","0","0","1","0","0","0","0","0","1","0","0","0","0","0","0","0","0","0","0","0","1","1","0","0
","0","0","0","1","1","1","0","0","0","1","1","0","0","0","0","0","0","120","3","0","NID","tested_
positive"

"64","80","70","17.5","50","1","1","0","0","0","0","0","1","0","0","0","0","0","0","0","0","0","0"
,"0","0","0","0","0","0","0","0","0","1","0","1","0","0","0","0","0","0","0","0","0","0","0","0","0"
,"0","0","0","0","0","0","0","0","0","0","0","0","0","0","0","0","0","0","0","0","0","0","0","0","0"
,"0","0","0","0","0","0","0","0","0","0","0","0","0","0","0","0","0","14","6","0","NID","tested_ne
gative"

"60","75","80","25","75","1","1","0","1","0","0","1","0","0","0","1","0","1","1","1","0","0","0","
0","0","0","1","0","0","0","0","0","1","1","1","0","0","0","0","0","1","0","0","0","1","1","1","0","
1","0","0","0","0","1","0","0","1","1","0","0","0","0","0","0","0","0","1","0","0","1","0","0","0","
0","0","0","0","1","1","0","0","0","1","0","0","1","1","0","0","0","21","6","0","NID","tested_neg
ative"

"43","116","84","21","70","1","1","1","1","0","0","1","0","0","0","0","0","1","1","0","1","0","1",
"0","0","0","0","0","0","0","0","0","1","1","0","0","0","0","0","0","1","0","1","0","0","1","1","0",

"0","0","0","1","0","1","0","0","0","1","0","0","0","0","0","1","0","0","0","0","1","1","0","0","0",
"0","0","0","0","1","0","0","0","0","1","0","1","0","0","0","0","1","14","5","0","GTD","tested_po
sitive"

"24","109","90","19.5","59","1","1","1","1","0","1","0","0","0","0","1","0","0","1","1","1","0","1
","0","0","0","0","0","0","0","0","0","1","1","0","0","0","0","1","1","1","0","1","1","1","1","1","0
","0","0","0","1","0","1","0","0","0","1","0","0","0","0","0","0","0","0","0","0","1","1","0","0","0
","0","0","0","0","1","1","0","0","0","1","0","0","0","0","0","1","0","7","3","1","IND","tested_po
sitive"

"27","132","75","22","65","1","1","1","1","1","0","0","1","1","0","0","1","1","1","1","1","0","0",
"0","1","0","1","0","0","1","0","0","1","1","1","0","0","1","1","1","1","0","1","1","1","0","1","0",
"0","0","1","1","1","1","0","1","1","1","0","1","1","0","0","0","0","0","0","0","0","1","1","1","1",
"0","0","1","0","1","1","0","0","0","1","1","0","1","0","1","0","0","30","3","3","NID","tested_po
sitive"

"63","127","86","20","50","1","1","1","1","1","1","1","1","1","1","0","0","1","1","0","0","0","0",
"0","1","0","0","0","0","1","0","0","1","1","0","0","0","0","0","0","0","0","0","1","0","1","0",
"1","0","0","1","0","0","0","0","1","1","0","0","0","0","0","0","0","0","0","0","1","0","0","0",
"0","0","0","0","0","1","0","0","0","1","1","0","0","0","0","0","0","30","3","0","NID","tested_po
sitive"

"70","142","90","16","42","1","1","0","1","1","0","0","0","0","0","0","0","1","1","1","1","0","0",
"0","0","0","0","0","0","0","0","0","1","1","0","0","0","0","1","0","0","0","0","0","0","1","1","0",
"0","0","0","1","0","0","0","0","0","1","0","0","0","0","0","0","0","0","0","0","1","0","0","0",
"0","0","0","0","1","0","0","0","0","1","0","0","0","0","0","0","0","21","6","0","NID","tested_po
sitive"

"40","111","70","18.5","55","1","1","0","1","1","1","0","0","0","0","1","0","1","1","1","0","0","0
","0","0","0","0","0","0","0","0","0","1","1","1","0","0","0","0","0","1","0","0","0","0","1","1","0
","0","0","0","0","0","1","0","0","0","1","0","0","0","1","0","0","1","0","0","0","0","1","0","0","0
","0","0","0","0","1","0","0","0","0","1","0","0","0","0","0","0","0","7","1","0","NID","tested_po
sitive"

"27","70","80","19","52","1","1","0","0","0","1","1","0","0","0","1","0","1","1","1","0","0","0","
0","0","0","0","0","0","0","0","0","1","1","1","0","0","0","0","0","1","0","0","0","0","1","1","0","
0","0","0","0","0","1","0","0","0","1","0","0","0","1","0","0","1","0","0","0","0","1","0","0","0","
0","0","0","0","1","0","0","0","0","1","0","0","0","0","0","0","0","14","2","1","NID","tested_neg
ative"

"60","113","85","18","45","1","1","0","1","1","1","1","1","1","0","0","1","1","1","1","1","0","1",

"1","0","0","0","0","0","0","0","0","1","1","1","0","0","1","0","1","1","1","1","1","1","1","1","1",
"1","1","1","1","1","1","1","1","1","1","0","1","0","1","1","1","0","0","0","1","1","1","1","1","0",
"0","0","1","1","1","1","0","0","0","1","1","0","0","0","0","0","0","14","6","0","NID","tested_po
sitive"

"31","181","90","21","65","1","1","1","0","0","0","0","0","0","0","0","0","0","1","0","0","0","1",
"0","0","0","0","0","0","0","0","0","1","1","0","0","0","0","0","0","1","0","1","0","1","0","1","0",
"0","0","0","1","0","1","0","0","0","1","0","0","0","0","0","0","0","0","0","0","1","1","0","0","0",
"0","0","1","0","0","1","0","0","0","1","0","0","0","0","0","0","0","7","2","0","NID","tested_posi
tive"

"60","83","70","23","75","1","1","1","1","0","0","0","0","0","0","1","0","0","1","0","0","1","0","
0","0","0","0","0","0","0","0","0","1","1","0","0","0","0","0","1","0","0","0","0","1","1","1","0","
0","0","0","0","0","0","0","0","0","1","0","0","0","0","0","1","0","0","0","0","0","1","0","0","0","
0","0","0","0","1","0","0","0","0","1","1","1","0","0","0","0","1","21","5","1","NID","tested_neg
ative"

"50","147","80","27","98","1","1","1","1","0","0","0","0","0","0","0","0","1","1","0","0","0","0",
"0","0","1","0","0","0","0","0","0","1","1","1","0","0","0","0","0","0","0","0","0","0","1","1","0",
"0","0","0","1","0","0","0","0","0","1","0","0","0","0","0","0","1","0","0","0","0","1","0","0","0",
"0","0","0","0","1","0","0","0","0","1","0","1","0","0","0","0","1","30","6","5","NID","tested_po
sitive"

"73","84","100","19","61","1","1","1","1","1","1","1","0","0","0","1","1","1","1","1","1","1","1",
"0","0","0","0","1","0","1","1","1","1","1","1","1","1","0","0","0","1","1","1","0","1","1","1","1",
"1","0","0","1","1","1","1","1","0","1","1","0","0","0","0","0","0","0","0","1","1","1","0","0","0",
"1","0","0","0","1","1","0","0","0","1","0","0","0","0","0","0","0","21","2","0","IND","tested_ne
gative"

"55","140","90","24","86","1","1","1","1","0","0","0","0","0","0","1","1","0","1","0","0","0","0",
"0","0","0","0","0","0","0","0","0","1","1","1","0","0","0","0","0","0","0","1","1","1","1","1","0",
"0","0","0","1","0","0","0","0","0","1","0","0","0","0","0","0","0","0","0","0","0","1","0","0","0",
"0","0","0","0","1","1","0","0","0","1","1","0","0","0","0","0","0","30","6","2","NID","tested_po
sitive"

"50","132","80","25","79","1","1","1","1","0","0","0","0","0","0","1","1","1","1","0","0","0","0",
"0","0","0","0","0","0","0","0","0","1","1","0","0","0","0","1","0","0","0","0","0","1","1","1","0",
"0","0","0","1","0","0","0","0","0","1","0","0","0","0","0","0","0","0","0","0","0","1","0","0","0",
"0","0","0","0","1","1","0","0","0","1","0","0","0","0","0","0","0","30","2","1","NID","tested_po
sitive"

"55","120","92","23","70","1","1","1","1","0","0","0","0","0","0","1","0","0","1","0","0","0","0",
"0","0","0","0","0","0","0","0","0","1","1","0","0","0","0","1","1","1","1","1","1","1","1","1","0",
"0","0","0","1","0","1","1","0","0","1","0","1","0","0","0","0","0","0","1","0","0","1","0","0","0",
"0","0","0","0","1","1","0","0","0","1","1","0","0","0","0","0","0","30","3","0","NID","tested_po
sitive"

"60","109","85","19","58","1","1","1","1","0","0","0","0","0","0","1","1","0","1","0","0","0","0",
"0","0","0","0","0","0","0","0","0","1","1","0","0","0","0","0","0","0","0","1","1","1","1","1","0",
"0","0","0","1","0","0","0","0","0","1","0","0","0","0","0","0","0","0","0","0","0","1","0","0","0",
"0","0","0","0","1","1","0","0","0","1","0","0","0","0","0","0","0","28","6","2","NID","tested_po
sitive"

"40","136","90","35","105","1","1","1","0","0","0","0","0","0","0","1","0","0","1","0","0","0","0
","0","0","1","0","0","0","0","0","0","1","1","0","0","0","0","1","1","0","1","0","0","0","0","0","0
","0","0","0","0","0","0","1","0","0","0","0","0","0","0","0","0","0","0","0","0","0","0","0","0","0
","0","0","0","0","1","0","0","0","0","0","0","1","0","0","0","0","1","28","6","1","NID","tested_p
ositive"

"30","121","80","30.5","92","1","1","1","1","0","0","0","0","0","0","1","1","0","1","0","0","0","0
","0","0","0","0","0","0","0","0","0","1","1","0","1","0","0","0","0","1","0","0","0","0","1","1","0
","0","0","0","1","0","1","0","0","0","1","0","0","0","0","0","0","0","0","0","0","0","0","0","0","0
","0","0","0","0","1","0","0","0","0","1","0","1","0","0","0","0","1","30","3","0","NID","tested_p
ositive"

"56","77","70","26.6","83","1","1","1","0","0","0","0","0","0","0","1","0","0","0","0","0","0","0"
,"0","0","0","0","0","0","0","0","0","1","0","0","0","0","0","0","0","0","0","0","0","1","0","0","0"
,"0","0","0","0","0","0","0","0","0","0","0","0","0","0","0","0","0","0","0","0","0","0","0","0","0"
,"0","0","0","0","0","1","0","0","0","0","1","0","0","0","0","0","0","60","2","0","NID","tested_ne
gative"

"61","80","60","28","86","1","1","1","1","1","1","0","0","0","0","1","1","1","1","1","0","0","0","
0","1","0","0","0","0","0","0","0","1","1","1","0","0","0","0","0","0","0","0","0","0","1","1","0","
0","0","0","1","0","0","1","1","0","1","0","1","1","0","0","0","0","0","1","0","0","1","0","0","0","
1","0","0","1","1","0","0","0","0","1","0","1","0","0","0","0","1","30","5","0","NID","tested_neg
ative"

"38","90","70","23","95","1","1","1","1","0","0","0","0","0","0","0","0","1","0","0","0","1","
0","0","1","0","0","0","0","0","0","1","1","0","0","0","0","0","0","0","1","0","0","0","1","1","0","
0","0","0","0","0","0","1","0","0","1","0","0","0","0","0","0","0","0","0","0","0","1","0","0","0","
0","0","0","0","1","0","0","0","0","1","1","1","0","0","0","0","1","28","6","0","NID","tested_neg

ative"

"40","145","60","22","62","1","1","0","0","0","0","0","0","0","0","0","0","0","0","0","0","0","0",
"0","0","0","0","0","0","0","0","0","1","0","0","0","0","0","0","0","0","0","0","0","0","0","0","0",
"0","0","0","0","0","0","0","0","0","0","0","0","0","0","0","1","0","0","0","0","0","0","0","0","0",
"0","0","0","0","0","0","0","0","0","0","0","0","0","0","0","0","0","14","3","1","NID","tested_po
sitive"

"50","82","77","24","79","1","1","1","0","0","0","0","0","0","0","1","0","0","0","0","0","0","0","
0","0","0","0","0","0","0","0","0","1","0","0","0","0","0","0","0","0","0","0","0","0","0","0","0","
0","0","0","0","0","0","0","0","0","0","0","0","0","0","0","0","0","0","0","0","0","0","0","0","0","
0","0","0","0","0","0","0","0","0","0","0","1","0","0","0","0","1","60","3","2","NID","tested_neg
ative"

"47","133","91","18.5","61","1","1","1","0","0","0","0","0","0","0","0","0","0","0","0","0","0","0
","0","0","0","0","0","0","0","0","0","1","0","0","0","0","0","0","0","0","1","0","1","1","0","0","0
","0","0","0","1","0","0","1","0","0","0","0","0","0","0","0","0","0","1","0","0","0","0","0","0
","0","0","0","0","0","1","0","0","0","0","0","0","0","0","0","0","0","14","9","3","GTD","tested_
positive"

"59","151","80","18","62","1","1","1","1","1","0","0","0","0","0","0","0","1","1","1","1","0","1",
"0","0","0","0","0","0","0","0","0","1","1","0","0","0","0","0","0","1","0","0","0","1","1","1","0",
"0","0","0","0","0","1","0","0","0","1","0","0","0","0","0","0","0","0","0","0","0","1","0","0","0",
"0","0","0","0","1","1","0","0","0","1","0","0","0","0","0","0","0","30","5","0","NID","tested_po
sitive"

"63","174","89","24","89","1","1","1","0","0","0","0","0","0","0","0","0","0","0","0","0","0","0",
"0","0","0","0","0","0","0","0","0","1","0","0","0","0","0","0","0","0","0","0","0","0","0","0","0",
"0","0","0","1","0","0","0","1","0","0","1","0","0","0","0","0","0","0","1","0","0","0","0","0","0",
"0","0","0","0","0","0","0","0","0","0","0","1","0","0","0","0","1","30","6","0","NID","tested_po
sitive"

"45","65","70","22","73","1","1","0","0","0","0","0","0","0","0","0","0","0","0","0","0","0","0","
0","0","0","0","0","0","0","0","0","1","0","0","0","0","0","1","0","0","0","0","0","0","0","0","0","
0","0","0","0","0","0","0","0","0","0","0","0","0","0","0","0","0","0","1","0","0","0","0","0","0","
0","0","0","0","0","0","0","0","0","0","0","0","0","0","0","0","0","90","2","1","NID","tested_neg
ative"

"68","193","90","27","73","1","1","1","1","1","1","0","0","0","0","0","0","1","1","0","0","0",
"0","0","0","0","0","0","0","1","0","1","1","1","1","1","0","1","1","1","0","0","0","0","1","1","1",
"0","0","0","1","0","1","1","0","1","1","1","0","0","0","0","0","0","1","1","1","0","0","0","0","0",

"0","0","0","0","1","1","0","0","0","0","0","0","0","1","0","0","0","30","6","3","NID","tested_po
sitive"

"71","211","80","19","60","1","1","1","0","0","0","0","0","0","0","0","0","0","0","0","0","0","0",
"0","0","0","0","0","0","0","0","0","0","0","0","0","0","0","1","0","0","0","0","0","1","0","0","0",
"0","0","1","0","0","0","0","0","0","0","1","1","0","0","0","0","0","0","1","1","0","0","0","0","0",
"0","0","0","0","0","1","0","0","0","0","0","0","0","1","0","0","0","30","3","1","NID","tested_po
sitive"

"49","101","55","21.5","65","1","1","1","0","0","0","0","0","0","1","0","0","0","0","0","0","0
","0","0","0","0","0","0","0","0","0","1","0","0","0","0","0","0","0","1","0","0","1","0","0","0","0
","0","0","0","0","0","0","0","0","0","0","0","0","0","0","0","1","0","0","0","0","0","0","0","0
","0","0","0","0","0","0","0","0","0","0","0","0","0","0","0","0","0","30","6","3","NID","tested_n
egative"

"59","91","78","27","78","1","1","0","0","0","0","0","0","0","0","0","0","0","0","0","0","0","0","
0","0","0","0","0","0","0","0","0","1","0","0","0","0","0","0","0","0","0","0","0","0","0","0","0","
0","0","0","0","0","0","0","0","0","0","0","0","0","0","0","0","0","0","0","0","0","0","0","0","0","
0","0","0","0","0","0","0","0","0","0","0","0","0","0","0","0","0","28","6","1","NID","tested_neg
ative"

"45","156","90","22.5","68","1","1","1","1","0","0","0","0","0","0","1","0","0","1","1","0","0","1
","1","1","0","0","0","0","0","0","0","1","1","0","0","0","0","1","0","1","0","1","1","1","1","1","0
","0","0","1","1","0","1","0","0","0","1","0","0","0","0","0","0","0","0","0","1","1","1","1","0","0
","1","1","1","0","1","1","0","0","1","1","1","0","0","0","0","0","0","21","2","1","NID","tested_p
ositive"

"14","80","50","15","31","1","1","1","1","0","0","0","0","0","0","0","0","1","1","0","1","1","0","
1","0","1","1","1","1","0","0","0","1","1","0","0","0","0","1","0","1","0","0","1","1","1","1","0","
0","0","0","0","0","0","0","1","1","1","0","0","0","0","0","0","0","0","1","1","1","1","1","0","0","
0","0","0","0","1","1","0","0","0","0","1","1","0","0","0","0","0","14","6","2","GTD","tested_ne
gative"

"58","201","80","19","74","1","1","1","1","1","1","0","0","0","0","0","0","0","1","1","0","0","0",
"0","0","0","0","0","0","0","1","0","1","1","1","1","1","0","1","1","1","0","0","0","0","1","1","1",
"0","0","0","1","0","1","1","0","1","1","1","0","0","0","0","0","0","1","1","1","0","0","0","0","0",
"0","0","0","0","1","1","0","0","0","0","0","0","0","1","0","0","0","30","6","3","NID","tested_po
sitive"

"61","145","90","18","57","1","1","1","0","0","0","0","0","0","0","0","0","0","0","0","0","0","0",
"0","0","0","0","0","0","0","0","0","0","0","0","0","0","0","1","0","0","0","0","0","1","0","0","0",

"0","0","1","0","0","0","0","0","0","0","1","1","0","0","0","0","0","0","1","1","0","0","0","0","0",
"0","0","0","0","0","1","0","0","0","0","0","0","0","1","0","0","0","30","3","1","NID","tested_po
sitive"

"42","72","60","22","64","1","1","1","0","0","0","0","0","0","0","1","0","0","0","0","0","0","0","
0","0","0","0","0","0","0","0","0","1","0","0","0","0","0","0","0","1","0","0","1","0","0","0","0","
0","0","0","0","0","0","0","0","0","0","0","0","0","0","0","1","0","0","0","0","0","0","0","0","
0","0","0","0","0","0","0","0","0","0","0","0","0","0","0","0","0","30","6","3","NID","tested_neg
ative"

"32","223","90","17.5","58","1","1","1","1","1","0","0","0","0","0","1","0","1","1","1","0","0","0
","0","0","0","0","0","0","0","0","0","1","1","1","0","0","0","0","0","1","0","0","1","1","1","1","0
","0","0","0","1","0","1","0","0","0","1","0","0","1","0","0","0","0","0","0","1","1","0","0","1
","0","0","1","0","0","1","0","0","0","1","1","0","0","0","0","0","0","56","2","0","NID","tested_p
ositive"

"69","138","80","23","65","1","1","1","1","1","0","0","0","1","0","1","0","1","1","1","0","0","0",
"0","0","0","0","0","0","0","0","0","1","1","1","0","0","0","0","0","1","0","0","0","1","1","1","0",
"0","0","0","1","0","1","0","0","0","1","0","0","0","0","0","0","0","0","0","0","0","1","0","0","0",
"0","0","0","0","1","1","0","0","0","1","1","0","0","0","0","0","0","60","2","0","NID","tested_po
sitive"

"62","175","70","25","72","1","1","1","0","1","0","1","1","1","1","1","1","1","1","1","1","0","1",
"0","0","0","0","0","0","0","0","0","1","1","0","0","0","0","0","0","1","0","0","0","1","1","1","0",
"0","0","0","1","0","1","0","0","0","1","0","1","0","0","0","0","0","0","0","0","1","0","0","1",
"0","0","0","0","1","1","0","0","0","1","1","0","0","0","0","0","0","28","3","0","NID","tested_po
sitive"

"41","119","90","28","85","1","1","1","0","0","0","0","0","0","0","1","0","1","1","1","1","0","0",
"0","0","0","0","0","0","0","0","0","1","1","1","0","0","0","0","0","1","0","0","0","1","1","0","0",
"0","1","1","1","0","1","0","0","0","0","0","1","0","0","0","0","0","0","0","1","1","1","1","0","0",
"0","0","1","0","1","0","0","0","1","1","1","1","0","0","0","0","1","56","3","0","NID","tested_po
sitive"

"71","76","55","20","58","1","1","1","0","0","0","0","1","0","0","1","1","1","1","1","0","1","0","
0","0","0","1","1","0","0","1","0","1","1","0","1","0","0","0","0","0","0","1","0","1","1","1","1","
0","1","0","0","1","0","0","0","1","1","0","0","0","0","0","1","0","0","0","0","0","1","1","0","0","
0","0","1","0","1","0","0","0","0","1","0","0","0","0","0","0","0","90","2","0","NID","tested_neg
ative"

"68","121","86","24","71","1","1","0","0","1","0","1","1","0","0","1","1","1","1","1","1","0","1",

"1","0","1","1","0","0","0","0","0","1","1","1","0","0","0","0","0","1","1","1","1","1","1","1","0",
"0","1","0","1","0","1","1","1","1","1","0","1","0","0","0","1","1","0","0","0","1","1","1","0","0",
"0","0","0","0","1","0","0","0","0","1","0","0","0","0","0","0","0","7","4","4","NID","tested_posi
tive"

"53","89","70","28","82","1","1","0","0","0","0","1","1","0","0","1","1","0","1","1","1","0","1","
1","0","0","1","0","0","0","1","0","1","1","0","1","0","0","0","0","0","0","0","0","1","1","1","
0","1","0","0","0","0","0","0","1","1","0","0","0","0","0","1","0","0","0","0","1","1","1","0","0","
0","0","0","0","1","0","0","0","0","1","0","0","0","0","0","0","0","90","1","0","NID","tested_neg
ative"

"31","215","90","19","65","1","1","0","1","0","0","0","1","1","1","0","0","0","1","1","1","0","1",
"0","0","0","1","0","0","0","0","0","1","1","1","0","0","0","0","0","0","0","1","0","0","1","1","0",
"0","1","0","1","0","0","0","1","0","1","0","0","0","0","0","0","0","0","0","0","0","1","1","0","0",
"0","0","1","0","1","0","0","0","0","1","1","0","0","0","0","0","0","30","4","2","GTD","tested_po
sitive"

"43","177","65","23","75","1","1","1","0","0","0","0","0","0","0","0","0","0","0","0","0","0","0",
"0","0","0","1","0","0","0","0","0","1","1","1","0","0","0","0","0","0","1","0","0","0","1","1","0",
"0","1","0","0","0","0","1","0","0","1","0","0","0","0","0","0","0","0","0","0","0","1","1","0","0",
"0","0","0","0","1","0","0","0","0","1","0","1","0","0","0","0","1","14","6","6","GTD","tested_po
sitive"

"36","181","80","22","65","1","1","0","1","1","0","0","1","1","1","1","1","1","1","1","1","1","1",
"1","0","1","1","1","1","0","1","1","1","1","1","1","1","1","0","0","0","1","1","1","0","1","1","1",
"1","1","0","0","1","0","1","1","1","1","0","0","1","0","0","1","1","0","0","0","1","1","1","0","0",
"1","1","1","0","1","1","1","1","0","1","0","0","0","1","0","0","0","42","3","1","NID","tested_po
sitive"

"61","155","100","23","69","1","1","0","1","1","0","0","1","0","0","0","0","1","1","1","1","0","0
","0","0","1","0","0","0","0","0","0","1","1","0","0","0","0","0","0","0","1","0","0","0","1","1","0
","0","0","0","1","0","0","1","0","1","1","0","0","1","0","0","0","1","1","1","0","0","1","0","0","0
","0","0","1","0","1","0","0","0","0","1","0","0","0","0","0","0","0","7","5","0","NID","tested_po
sitive"

"64","118","80","18.5","58","1","1","0","1","1","1","1","1","0","0","1","1","1","1","1","1","0","0
","0","1","0","1","0","0","0","0","0","1","1","0","0","0","0","0","0","0","0","0","0","0","1","1","0
","0","1","0","1","0","0","0","0","1","1","0","0","0","0","0","1","0","0","0","0","0","1","1","0","0
","0","0","1","0","1","0","0","0","0","1","1","0","0","0","0","0","0","14","4","4","NID","tested_p
ositive"

"61","129","90","28","89","0","0","0","0","0","0","0","0","0","0","0","0","1","0","0","0","0",
"0","0","0","0","0","0","0","0","0","0","1","1","0","0","0","0","1","0","0","0","0","1","1","0","0",
"0","0","0","0","0","0","0","0","0","0","0","0","0","0","0","0","0","0","0","0","1","1","0","0","0",
"0","0","0","0","0","0","0","0","0","0","0","0","0","0","0","0","0","49","3","0","NID","tested_po
sitive"

"64","210","80","20.5","60","0","1","0","0","1","0","0","0","0","0","0","0","1","1","0","0","0","0
","0","0","0","0","0","0","0","0","0","0","0","1","0","0","0","0","0","0","0","0","0","0","0","1","0
","0","0","0","0","0","0","0","0","0","1","1","0","0","0","0","0","0","0","0","0","0","0","0","0","0
","0","0","0","0","1","0","0","0","0","0","1","0","0","0","0","0","0","7","2","0","NID","tested_po
sitive"

"57","95","70","22","62","0","1","1","1","1","1","0","0","1","0","0","0","1","1","0","0","0","0","
1","1","0","0","0","0","0","0","0","1","1","1","0","0","0","0","0","0","1","0","0","1","1","1","0","
0","0","0","0","0","0","1","0","0","1","1","0","0","0","0","1","0","0","1","0","0","1","0","0","0","
0","1","0","0","1","1","1","0","0","1","1","0","0","0","0","0","0","90","2","0","NID","tested_neg
ative"

"61","136","85","26","80","0","1","1","0","0","0","0","0","0","0","0","0","0","0","0","0","0","1",
"0","0","0","0","0","0","0","0","0","1","0","1","0","0","0","0","0","0","0","0","0","1","1","1","0",
"0","0","0","1","0","0","0","0","0","1","0","0","0","0","0","0","0","0","0","0","0","1","0","0","0",
"0","0","0","0","1","1","0","0","0","1","1","0","0","0","0","1","0","28","2","0","IND","tested_po
sitive"

"59","119","92","25","70","0","0","0","0","0","0","0","1","0","0","0","0","0","0","0","0","1","0",
"0","0","1","0","0","0","0","0","0","0","1","1","0","0","0","0","0","0","0","0","0","0","1","0","0",
"0","0","0","1","0","0","0","0","0","0","0","0","0","0","0","0","0","0","0","0","0","0","0","0","0",
"0","0","0","0","0","0","0","0","0","0","0","0","0","0","0","0","0","35","4","0","NID","tested_po
sitive"

"40","75","70","20","57","0","0","0","0","0","0","0","1","0","0","0","0","0","1","0","0","0","0","
0","0","0","0","0","0","1","0","0","0","1","0","0","0","0","0","0","0","0","0","1","0","1","0","0","
0","0","0","0","0","0","0","0","0","0","0","0","0","0","0","0","0","0","0","0","0","0","0","0","0","
0","0","0","0","0","0","0","0","0","0","0","0","0","0","0","0","0","28","3","0","NID","tested_neg
ative"

"44","160","90","35","104","0","1","0","1","1","1","1","1","1","0","0","1","0","1","0","0","0","1
","0","1","1","0","0","0","0","0","0","1","1","1","0","0","0","1","0","0","1","0","1","0","1","0","0
","0","0","1","1","1","0","1","0","0","0","0","0","0","0","0","0","0","0","0","0","0","0","0","0","0
","0","0","0","0","0","0","0","0","0","0","1","1","0","0","0","0","1","14","3","0","NID","tested_p

ositive"

"46","133","80","18","48","0","0","1","0","1","1","0","0","1","0","1","0","1","1","1","1","0","1",
"1","0","0","0","0","1","1","0","0","0","1","1","0","0","0","0","0","0","0","1","0","1","1","1","0",
"0","0","0","1","0","0","0","0","0","0","0","0","0","0","0","0","0","1","1","0","0","0","0","0","1",
"0","1","0","0","0","1","0","0","0","0","1","0","0","0","0","0","0","7","4","0","IND","tested_posi
tive"

"52","154","84","29","89","0","0","0","0","0","0","0","0","0","0","1","0","0","0","0","0","1","0",
"0","0","0","0","0","0","0","0","0","0","0","1","0","0","0","0","1","1","0","0","0","0","0","0","0",
"0","0","0","1","0","1","0","0","0","0","0","0","0","0","0","0","0","0","0","0","0","0","0","0","0",
"1","1","0","0","0","0","0","0","0","0","0","0","0","0","0","0","0","70","2","0","NID","tested_po
sitive"

"64","84","70","21","60","0","1","1","1","1","0","0","1","0","0","0","0","1","0","0","0","0","0","
0","0","0","0","0","0","0","0","0","1","0","1","0","0","0","0","0","0","0","0","0","1","1","1","0","
0","0","0","0","0","0","0","0","0","1","0","0","0","0","0","0","0","0","0","0","1","1","0","0","0","
0","0","0","0","0","0","0","0","0","1","0","0","0","0","0","0","0","84","2","0","NID","tested_neg
ative"

"52","140","60","19","61","1","1","1","1","1","1","1","1","1","1","1","1","1","1","0","1","1","1",
"1","1","1","1","1","1","1","1","1","1","1","1","1","1","0","0","1","1","1","1","1","1","1","1","1",
"1","0","0","0","1","1","1","1","0","0","0","0","0","0","0","1","0","0","0","0","1","0","0","0","0",
"1","1","1","1","0","1","0","0","0","1","0","0","0","0","0","0","0","90","1","0","NID","tested_po
sitive"

"47","90","50","21","57","1","0","1","0","0","0","0","1","1","0","1","0","0","1","1","1","1","1","
1","1","0","1","0","0","0","0","0","0","1","1","0","0","0","0","1","1","0","1","1","1","1","0","0","
0","0","0","0","0","1","0","1","0","0","0","0","0","0","0","0","0","0","0","0","1","0","0","0","0","
1","1","0","0","0","1","0","0","0","0","1","0","1","0","0","0","0","28","3","0","NID","tested_neg
ative"

"57","170","90","22","58","0","1","1","1","1","1","0","1","0","0","0","0","1","1","1","1","1","1",
"1","1","0","0","0","0","0","0","0","1","1","1","0","0","0","0","0","1","0","1","1","1","1","0","0",
"0","0","0","1","1","1","0","0","0","0","1","0","0","0","0","1","0","0","1","0","1","0","0","0","0",
"1","1","0","0","0","1","0","0","0","0","1","0","0","0","0","0","0","70","4","0","NID","tested_po
sitive"

"48","132","60","18.5","51","1","1","1","0","1","1","0","1","1","1","0","0","1","1","1","1","0","0
","1","1","0","0","0","0","0","0","0","1","1","1","0","0","0","0","0","0","0","0","1","1","1","1","0
","0","0","0","0","0","1","0","0","0","0","0","0","0","0","0","0","0","0","0","0","0","0","0","0","1

","0","0","1","0","0","0","0","0","0","1","0","0","0","0","0","0","0","14","4","0","NID","tested_positive"

"51","65","40","21","58","0","0","1","0","0","0","0","1","0","0","0","0","0","1","1","0","1","1","1","0","0","0","0","0","0","0","0","0","1","1","0","0","0","0","0","0","0","0","0","1","1","0","0","0","0","0","0","0","0","0","0","0","0","0","0","0","0","0","0","0","0","0","0","0","0","0","0","1","0","0","1","0","0","0","0","0","0","0","1","0","0","0","0","0","0","70","2","0","NID","tested_negative"

"54","120","90","18","52","0","0","0","0","0","0","0","0","0","0","0","0","0","0","0","0","0","0","0","0","0","0","0","0","0","0","0","0","0","0","0","0","0","0","0","0","0","0","0","0","1","0","0","0","0","0","0","1","0","0","0","0","0","0","0","0","0","0","0","0","0","0","0","0","0","0","0","0","0","0","0","0","0","0","0","0","0","0","0","0","0","0","0","0","0","0","0","42","2","0","NID","tested_positive"

"45","140","80","19","92","1","1","1","0","1","1","0","1","0","0","1","0","1","1","1","1","1","1","0","1","0","0","0","0","0","0","0","1","1","1","0","1","0","1","1","0","0","0","0","0","0","1","0","0","1","1","1","1","0","0","0","0","0","0","0","0","0","0","0","0","0","0","1","1","1","1","0","0","1","1","1","0","0","0","0","0","0","0","1","1","0","0","0","0","1","28","1","0","IND","tested_positive"

"41","118","80","20","58","0","0","1","0","1","0","0","0","1","0","1","0","1","1","0","0","1","1","0","0","0","0","0","0","0","0","0","0","1","1","0","0","0","0","1","0","0","0","0","1","1","1","0","0","0","0","0","0","0","0","0","0","0","0","0","0","0","1","0","0","0","0","0","0","0","0","0","0","1","0","0","0","1","0","0","0","0","0","0","0","0","0","0","0","70","3","0","NID","tested_positive"

"39","171","70","19.5","58","0","0","1","0","1","0","0","0","0","0","1","0","1","1","0","1","0","1","1","0","0","0","0","1","1","0","0","0","1","0","0","0","0","0","1","0","0","0","0","1","1","1","0","1","0","0","0","0","0","0","0","0","0","0","0","0","0","0","0","0","0","0","0","0","0","1","0","0","0","0","1","1","0","1","0","0","0","0","0","1","0","0","0","0","0","0","28","2","0","NID","tested_positive"

"35","98","110","22","60","0","0","1","0","1","0","0","0","0","0","1","0","1","0","1","1","0","1","1","0","0","0","0","0","0","0","0","0","1","0","0","1","0","0","1","0","0","0","0","1","1","0","0","0","0","1","0","0","0","0","0","0","0","0","0","0","0","0","0","0","0","0","0","1","1","0","0","0","0","0","1","1","0","0","0","0","0","0","0","0","0","0","0","0","0","0","90","1","0","NID","tested_positive"

"32","125","86","20.5","60","1","0","1","1","1","1","0","1","1","1","0","1","1","1","1","1","1","1","1","1","0","0","0","0","0","1","0","1","1","1","1","1","0","1","0","1","1","1","1","1","1","1","0

","0","1","1","1","1","1","1","1","0","1","0","0","0","0","0","0","0","0","0","1","1","1","0","0","0
","1","1","1","0","1","1","0","0","0","0","1","0","0","0","0","0","0","7","6","0","NID","tested_po
sitive"

"50","81","60","19","48","0","0","1","0","1","0","0","1","0","0","1","0","1","1","1","1","1","0","
1","0","0","0","0","0","0","0","0","1","1","0","0","0","0","0","1","0","0","1","0","1","1","1","0","
0","0","0","0","0","0","0","0","0","1","0","0","0","0","0","0","0","0","0","0","1","0","0","0","0","
1","1","0","0","0","0","0","0","0","1","1","0","0","0","0","0","0","35","2","0","NID","tested_neg
ative"

"57","211","80","24","73","0","1","0","0","1","0","0","1","1","0","1","1","1","1","0","1","0","1",
"1","0","0","0","0","0","0","0","0","1","0","1","0","1","0","0","0","0","0","0","1","0","1","0","0",
"0","0","0","1","0","0","0","0","0","0","0","0","0","1","0","0","0","0","1","1","0","1","0","0","1",
"0","1","0","0","1","0","0","0","0","1","0","0","0","0","0","0","0","7","3","0","NID","tested_posi
tive"

"65","65","80","19.5","49","0","1","1","0","1","0","0","1","0","0","0","0","1","0","1","0","1","1"
,"0","0","0","0","0","0","0","0","0","1","1","1","0","0","0","0","0","0","0","0","0","1","0","0"
,"0","0","0","0","0","0","0","0","0","0","1","0","0","1","0","0","0","0","0","0","0","0","0","0"
,"1","0","0","0","0","0","0","0","0","0","0","0","0","0","0","0","0","70","2","0","NID","tested_ne
gative"

"45","50","60","23","65","0","1","1","0","1","1","0","1","0","1","1","1","1","1","1","1","0","0","
1","0","0","0","0","0","1","0","0","1","1","1","1","0","0","0","1","0","0","1","1","1","1","0","1","
0","1","0","0","0","0","0","0","0","0","1","0","0","0","0","1","0","0","0","0","1","0","0","0","0","
1","1","0","0","0","1","0","0","0","0","0","0","0","0","0","0","0","56","2","0","NID","tested_neg
ative"

"68","98","105","18","72","0","1","0","0","1","0","0","1","0","0","1","0","1","0","1","1","1","0",
"0","0","0","0","0","0","0","0","0","1","1","1","0","0","0","0","1","0","0","0","1","0","1","0","0",
"0","0","0","0","0","0","0","0","0","0","1","0","0","0","0","1","0","0","1","0","0","0","0","0","0",
"1","0","0","0","0","0","0","0","0","1","0","0","0","0","0","0","0","28","2","0","NID","tested_po
sitive"

"71","120","90","21.5","63","0","0","0","0","0","0","0","0","0","0","1","0","0","0","0","0","0","0
","0","0","0","0","0","0","0","0","0","0","0","1","0","0","0","0","0","0","0","0","0","0","0","0","0
","0","0","0","1","0","0","0","0","0","0","0","0","0","0","0","0","0","0","0","0","0","0","0","0","0
","0","0","0","0","0","0","0","0","0","0","0","0","0","0","0","0","0","21","5","0","NID","tested_p
ositive"

"53","112","100","19.5","94","0","1","0","0","1","0","0","1","0","0","1","0","1","0","1","0","1","

1","0","0","0","0","0","0","0","0","0","1","0","1","0","0","0","0","1","0","0","0","0","0","0","0","
0","0","0","0","1","0","0","0","0","0","0","0","0","0","0","0","0","0","0","0","0","0","0","0","0","
0","0","0","0","0","0","1","0","0","0","0","0","0","0","0","0","0","0","28","4","3","NID","tested_
positive"

"51","58","70","20","58","1","1","1","0","1","0","0","1","0","0","1","0","1","1","1","1","1","0","
0","0","0","0","0","0","0","0","0","1","1","0","0","0","0","0","1","0","0","0","1","1","1","0","0","
0","0","0","0","0","0","0","0","0","0","0","0","0","0","0","0","0","0","0","0","0","0","0","0","0","
1","1","1","0","0","1","0","0","0","0","1","0","0","0","0","0","0","14","8","0","NID","tested_neg
ative"

"61","128","94","19.5","56","1","1","1","0","1","0","0","1","0","0","0","1","1","1","1","1","1","1
","1","0","0","1","0","1","1","1","1","1","1","1","0","0","0","1","1","0","0","0","0","1","1","0","0
","0","0","1","1","1","1","0","0","0","0","1","0","0","0","0","1","0","0","0","1","1","1","0","0","1
","1","0","1","1","0","0","0","0","1","0","1","0","0","0","0","0","0","28","3","0","NID","tested_p
ositive"

"56","90","40","22","62","1","1","1","1","1","1","0","0","0","0","1","1","1","0","1","1","0","1","
1","1","1","0","0","1","1","0","0","1","1","0","0","0","0","0","1","0","0","0","0","1","1","0","0","
0","0","0","0","0","0","0","0","0","0","1","0","0","0","0","1","0","0","0","0","0","0","0","0","0","
1","0","1","0","0","1","0","0","0","0","0","0","0","0","0","0","0","28","3","0","NID","tested_neg
ative"

"40","68","70","28","80","0","0","1","0","0","0","0","0","0","0","0","0","0","0","0","0","0","0","
0","0","0","0","0","0","0","0","0","0","0","0","0","0","0","0","0","0","0","0","1","0","0","0","0","
0","0","0","0","0","0","0","0","0","0","0","0","0","0","0","0","0","0","0","0","0","0","0","0","0","
0","0","0","0","0","0","0","0","0","0","0","0","0","0","0","0","0","70","2","0","NID","tested_neg
ative"

"64","58","80","23","60","0","0","0","0","0","0","0","1","0","0","0","0","0","0","0","0","0","0","
0","0","0","0","0","0","0","0","0","0","0","1","0","0","0","0","0","0","0","0","0","0","1","0","0","
0","0","0","0","0","0","0","0","0","0","0","0","0","0","0","0","0","0","0","0","0","0","0","0","0","
0","0","0","0","0","0","0","0","0","0","0","0","0","0","0","0","0","28","6","0","NID","tested_neg
ative"

"38","177","60","18","52","1","1","1","0","0","0","0","1","1","1","1","1","0","1","1","0","1","1",
"1","1","1","0","0","1","1","0","0","1","1","1","0","1","0","0","1","0","1","1","1","1","1","1","0",
"0","1","0","0","0","1","0","0","1","0","0","0","0","0","0","0","0","0","0","0","1","0","0","0","0",
"1","1","0","0","0","1","0","0","0","0","1","0","0","0","0","0","0","7","5","0","NID","tested_posi
tive"

"60","158","90","19.5","79","1","1","1","1","1","0","1","1","1","0","1","0","1","1","1","1","1","1
","0","0","0","0","0","0","0","0","0","1","1","1","0","0","0","0","0","1","0","0","0","1","1","0","0
","0","0","0","1","0","1","0","0","0","0","1","0","0","0","0","0","0","0","0","0","0","0","0","0","1
","1","1","1","0","0","1","0","0","0","0","1","0","0","0","0","0","0","70","1","0","NID","tested_p
ositive"

"51","40","110","21","65","1","1","1","0","0","0","0","0","0","0","1","0","0","1","1","1","0","1",
"0","0","1","0","0","1","1","0","0","1","1","1","0","0","0","0","0","1","1","1","1","1","1","0","0",
"0","0","1","0","0","1","1","0","0","0","0","0","0","0","0","0","0","0","0","0","0","0","0","0","0",
"0","1","0","0","0","1","0","0","0","0","1","0","0","0","0","0","0","90","2","0","NID","tested_ne
gative"

"43","135","87","17","65","1","1","1","0","1","0","1","0","0","0","1","0","1","1","0","0","0","1",
"0","0","0","0","0","0","0","0","0","1","1","0","0","0","0","0","0","0","0","0","1","1","1","0","0",
"0","0","0","1","0","0","0","0","0","0","0","0","0","0","0","0","0","0","0","0","0","0","0","0","0",
"0","0","0","0","0","1","0","0","0","0","1","0","0","0","0","0","0","56","2","0","NID","tested_po
sitive"

"60","210","40","24.5","72","0","0","1","0","1","0","0","1","1","0","0","0","1","1","1","1","0","0
","0","0","0","0","0","0","0","0","0","0","1","1","0","0","0","0","1","0","0","0","1","1","0","0
","0","0","0","0","0","1","0","0","0","0","0","0","0","0","0","0","0","0","0","0","0","0","0","0","0
","0","0","0","0","0","0","0","0","0","0","1","0","0","0","0","0","0","14","8","0","NID","tested_p
ositive"

"76","144","80","18","58","1","1","1","0","1","0","0","1","0","0","1","0","1","1","1","1","0","1",
"0","0","0","0","0","1","1","0","0","1","1","1","0","0","0","0","0","1","0","0","1","1","1","0","0",
"0","0","0","0","0","1","0","0","0","0","1","1","1","0","0","0","0","0","0","0","1","0","0","0","1",
"0","0","0","0","0","1","0","0","0","0","1","0","0","0","0","1","0","28","3","0","IND","tested_po
sitive"

"53","188","90","26","78","0","1","1","1","1","1","1","0","0","0","0","1","1","1","0","0","0",
"1","0","0","0","0","0","0","0","0","0","0","0","0","0","0","0","0","0","0","0","0","0","0","0","0",
"0","0","0","0","0","0","0","0","0","0","1","0","0","1","1","0","0","0","0","0","0","0","0","0","0",
"0","0","0","0","0","0","0","0","0","0","0","0","0","0","0","0","0","77","2","1","NID","tested_po
sitive"

"50","168","80","17.5","65","0","0","1","0","0","0","0","0","0","0","0","0","0","0","0","0","0","0
","0","0","0","0","0","0","0","0","0","0","0","1","0","0","0","0","0","0","0","0","0","0","1","0","0
","0","0","0","0","0","0","0","0","0","0","0","0","0","0","0","0","0","0","0","0","0","0","0","0","0
","0","0","0","0","0","1","0","0","0","0","1","0","0","0","0","0","0","70","3","0","NID","tested_p

ositive"

"53","131","80","23","80","1","1","1","1","1","0","0","1","1","0","1","0","1","1","1","1","1","1",
"0","0","0","0","0","0","0","0","0","1","1","1","0","0","0","0","0","1","0","1","1","1","0","0","0",
"0","0","0","0","0","1","0","0","0","0","0","0","0","0","0","0","0","0","0","0","0","0","0","0","0",
"0","0","1","0","0","0","0","0","0","0","1","0","0","0","0","0","0","56","2","0","NID","tested_po
sitive"

"71","119","90","23","88","0","0","0","0","1","0","0","0","0","0","1","0","1","1","0","0","0","1",
"1","0","0","0","0","1","1","0","0","0","1","1","0","1","0","0","1","0","0","0","0","0","1","0","0",
"0","0","0","1","0","0","0","0","0","0","0","0","0","0","0","0","0","0","0","0","0","0","0","0","0",
"0","0","0","0","0","0","0","0","0","0","1","0","0","0","0","0","0","42","3","0","NID","tested_po
sitive"

"48","201","80","18.5","60","1","1","1","0","1","0","0","1","0","1","1","0","1","1","1","0","0","0
","0","0","0","0","0","0","0","0","0","1","1","0","0","0","0","0","0","0","0","0","1","1","0","0
","0","0","0","0","0","0","0","0","0","0","0","0","0","0","0","0","0","0","0","0","1","1","0","0","0
","0","0","0","0","0","1","0","0","0","0","1","0","0","0","0","0","0","70","2","0","NID","tested_p
ositive"

"72","66","90","23","65","0","0","1","0","0","0","0","0","1","0","0","0","1","1","0","0","1","0","
1","0","0","0","0","0","0","0","0","0","1","1","0","0","0","1","0","0","0","0","0","1","1","0","0","
0","0","0","1","0","0","0","0","0","0","0","0","0","0","0","0","0","0","0","0","0","0","0","0","1","
1","0","1","0","0","1","0","0","0","0","0","0","0","0","0","0","0","28","3","0","NID","tested_neg
ative"

"70","190","30","25","72","1","1","1","1","1","0","0","0","0","0","1","0","1","1","1","0","1","0",
"1","0","0","0","0","0","0","0","0","1","1","1","0","0","0","0","0","0","0","0","0","1","1","0","0",
"0","0","0","1","0","0","0","0","0","0","0","0","0","0","0","0","0","0","0","0","0","0","0","0","1",
"0","0","1","0","0","1","0","0","0","0","0","0","0","0","0","0","0","82","2","0","NID","tested_po
sitive"

"61","50","80","20","61","1","1","1","0","1","0","0","0","0","0","0","0","1","0","1","1","0","0","
0","0","0","0","0","0","0","0","0","1","0","0","0","0","0","0","0","0","0","0","0","1","0","0","0","
0","0","0","0","0","0","0","0","0","0","0","0","0","0","0","0","0","0","0","0","0","0","0","0","0","
0","0","0","0","0","1","0","0","0","0","1","0","0","0","0","0","0","77","2","0","NID","tested_neg
ative"

"46","115","100","17.5","85","1","1","1","1","1","0","0","1","1","1","1","1","1","1","1","1","1","
0","0","1","0","0","0","1","1","0","0","1","1","1","0","0","0","0","0","1","1","1","1","1","1","0","
0","0","0","0","1","0","1","1","0","0","0","1","0","0","0","0","0","0","0","0","0","1","0","0","0","

0","1","1","1","0","0","1","0","0","0","0","1","0","0","0","0","0","0","56","2","0","NID","tested_positive"

"62","119","87","22","70","0","0","0","0","0","0","0","0","0","0","0","0","0","0","0","0","0","1","0","0","0","0","0","0","0","0","0","0","0","0","0","0","0","0","0","0","0","1","0","0","0","0","0","0","0","0","1","0","0","0","0","0","0","0","0","0","0","0","0","0","0","0","0","0","0","0","0","0","0","0","0","0","0","0","0","0","0","0","0","0","0","0","0","0","0","56","1","0","NID","tested_positive"

"64","170","80","28","85","1","1","1","1","1","1","0","0","1","0","1","0","1","1","1","1","1","1","1","1","0","1","0","1","1","0","1","1","1","1","0","1","0","0","0","1","0","0","0","0","0","0","0","0","0","0","1","0","1","0","0","0","0","1","1","0","0","0","0","0","0","0","0","1","1","1","0","0","1","1","1","0","1","1","0","0","0","1","1","0","0","0","0","0","0","90","1","1","NID","tested_positive"

"47","118","90","18","55","1","0","0","0","0","0","1","1","0","0","0","0","0","1","0","1","0","0","0","0","0","1","0","0","0","0","0","0","1","0","0","0","0","0","0","0","0","0","0","0","0","0","0","0","0","0","0","0","0","0","0","0","0","0","0","0","0","0","0","0","0","0","0","0","0","0","0","0","0","0","0","0","0","0","0","0","0","0","0","0","0","1","0","0","0","0","0","0","28","2","0","NID","tested_positive"

"53","126","80","23","70","0","0","0","0","0","0","0","0","0","0","1","0","0","0","0","0","0","0","0","0","0","0","0","0","0","0","0","0","0","0","0","0","0","0","0","1","0","0","0","0","0","0","0","0","0","0","1","0","1","0","0","0","0","0","0","0","0","0","0","0","0","0","0","0","0","0","0","0","0","1","0","0","0","0","0","0","0","0","1","0","0","0","0","0","0","28","6","0","NID","tested_positive"

"50","111","90","19","85","0","0","1","0","1","0","0","0","1","1","0","0","0","1","0","0","0","0","1","0","0","0","0","0","0","0","0","0","1","1","0","1","0","0","0","0","0","0","0","1","1","0","0","0","0","0","1","0","0","0","0","0","0","0","0","0","0","0","0","0","0","0","0","0","0","0","0","0","0","0","1","0","0","0","1","0","0","0","0","1","0","0","0","0","0","0","7","4","1","NID","tested_positive"

"54","58","50","25","77","0","0","1","0","0","0","0","0","0","0","1","0","0","0","0","0","0","0","0","0","0","0","0","1","1","0","0","0","0","0","0","0","0","0","1","0","0","0","0","1","1","0","0","0","0","0","0","0","0","0","0","0","0","1","0","0","0","0","0","0","0","0","0","0","0","0","0","0","0","0","1","0","0","1","1","0","0","0","1","0","0","0","0","0","0","0","14","1","0","NID","tested_negative"

"37","98","110","20.5","61","1","0","1","0","0","0","0","0","0","0","0","0","1","0","0","0","0","0","0","0","1","0","0","0","0","0","0","1","0","0","0","0","0","0","1","0","0","1","1","1","0","0

","0","0","0","0","0","1","0","0","0","0","0","0","0","0","0","0","0","0","0","1","1","0","0","0
","0","0","0","0","1","1","0","0","1","0","0","0","0","0","0","0","0","7","5","0","NID","tested_po
sitive"

"56","211","60","16.5","48","1","1","1","0","1","0","0","1","1","0","1","1","1","1","1","0","1","1
","1","1","0","0","0","0","1","1","1","1","1","0","1","0","0","0","1","1","0","0","1","1","1","1","1
","1","0","0","0","1","1","0","1","0","1","1","0","0","1","0","0","0","0","1","0","0","0","0","0","0
","1","1","1","1","1","1","0","0","0","1","1","0","0","0","0","0","0","7","2","0","NID","tested_po
sitive"

"68","72","90","31","89","1","1","1","1","0","0","1","0","0","0","1","0","0","0","1","0","0","1","
0","0","1","1","0","1","0","0","0","1","1","0","0","0","0","0","1","1","1","0","1","1","1","1","0","
0","0","0","1","1","1","0","0","0","1","0","0","0","0","0","0","0","0","0","0","0","0","0","0","0","
0","0","0","0","1","1","0","0","1","1","0","0","0","0","0","0","0","360","0","0","NID","tested_ne
gative"

"38","54","60","19","59","0","1","0","0","0","0","0","0","0","0","0","0","0","0","0","1","0","0","
1","0","0","0","0","0","0","0","0","0","1","0","0","0","0","0","0","1","0","0","1","0","0","1","0","
0","0","0","0","1","1","0","0","0","1","0","0","0","0","0","0","0","0","0","0","0","1","0","0","0","
0","1","1","0","1","0","0","0","0","1","0","0","0","0","0","0","0","30","2","0","NID","tested_neg
ative"

"51","48","110","21","67","1","1","0","1","1","1","0","0","0","0","1","1","1","1","0","1","0","1",
"0","1","1","1","0","0","1","1","0","1","1","1","1","0","0","0","1","0","1","1","1","0","1","1","1",
"1","0","0","1","1","0","1","0","0","1","1","0","0","0","0","0","0","0","0","0","0","0","0","0","0",
"0","1","0","1","1","0","0","0","1","1","0","0","0","0","0","0","0","60","2","0","NID","tested_ne
gative"

"60","144","90","22","78","0","1","1","0","1","0","0","0","0","0","0","0","1","1","1","1","1","0",
"0","0","0","0","0","0","0","0","0","1","1","1","0","0","0","0","0","0","0","0","0","1","1","1","0",
"0","0","0","1","0","0","0","0","0","0","1","0","0","0","0","0","0","0","0","0","0","0","0","0","0",
"1","1","0","0","0","1","0","0","0","0","1","0","0","0","0","0","0","21","2","0","NID","tested_po
sitive"

"51","221","40","22.5","67","1","0","1","0","0","0","0","0","0","0","1","0","0","1","0","0","1","0
","0","0","0","0","0","0","0","0","0","0","1","1","0","0","0","0","0","0","0","0","1","1","0","0","0
","0","0","0","0","0","0","0","0","0","0","0","0","0","0","0","0","0","0","0","0","1","0","0","0","0
","0","1","1","0","0","1","0","0","0","0","1","0","0","0","0","0","0","63","3","0","NID","tested_p
ositive"

"32","60","70","30","82","1","1","0","0","0","0","0","0","1","0","1","0","0","1","1","0","0","0","

0","0","0","0","0","0","0","0","0","1","1","0","0","0","0","0","1","0","0","0","1","0","1","0","0","
1","0","0","0","0","0","0","0","0","0","0","1","1","0","0","0","0","0","0","0","1","1","0","0","0","
1","1","0","0","0","0","0","0","0","0","1","0","0","0","0","0","0","42","2","0","NID","tested_neg
ative"

"44","119","86","19","64","0","0","1","0","0","0","0","0","1","0","0","0","0","0","0","0","1","0",
"0","0","0","0","0","0","1","0","0","0","1","0","0","0","0","0","0","0","0","0","1","1","0","0","0",
"0","0","0","1","0","0","0","0","0","0","0","0","0","0","0","0","0","0","0","0","0","0","0","0","0",
"1","0","0","0","0","1","0","0","0","0","1","0","0","0","0","0","0","42","4","0","NID","tested_po
sitive"

"55","180","90","22","66","1","1","1","0","0","1","0","1","0","1","0","1","0","1","1","1","1","0",
"0","1","0","0","0","0","0","0","0","1","1","0","0","0","0","0","0","1","0","1","0","0","1","1","0",
"0","0","0","0","0","1","0","0","0","0","0","0","0","0","0","0","0","0","0","0","0","0","0","0","0",
"1","1","1","1","1","1","0","0","1","1","1","0","0","0","0","0","0","28","4","0","NID","tested_po
sitive"

"41","117","80","32","96","0","1","0","0","0","0","0","0","0","0","1","0","0","0","0","1","1","1",
"0","0","0","0","0","0","0","0","0","1","0","0","0","0","0","0","1","0","0","0","0","0","0","1","0",
"0","0","0","1","1","0","0","0","0","0","0","0","0","0","0","0","0","0","0","0","0","0","0","0","0",
"1","1","0","0","1","0","0","0","1","0","0","0","0","0","0","0","0","56","3","0","NID","tested_po
sitive"

"48","133","60","19","60","0","1","1","0","0","0","0","0","0","0","0","0","0","0","1","0","0","0",
"1","0","0","0","0","0","1","0","0","1","0","1","0","0","0","1","0","1","0","0","1","1","1","0","0",
"0","0","0","0","0","1","0","0","0","0","0","0","0","0","0","0","0","0","0","1","0","0","0","0","0",
"0","0","1","0","0","0","0","0","0","1","0","0","0","0","0","0","0","35","6","0","NID","tested_po
sitive"

"64","141","50","18","51","1","1","1","1","1","1","0","1","0","0","1","1","1","1","1","1","1","0",
"0","0","0","0","0","1","1","0","0","1","1","1","0","0","0","0","1","1","0","0","0","1","1","0","0",
"0","0","0","0","1","1","0","0","0","1","0","0","0","0","0","0","0","0","0","0","0","1","0","0","1",
"1","0","1","0","0","1","0","0","0","1","1","0","0","0","0","0","0","14","4","0","NID","tested_po
sitive"

"77","123","90","16.5","47","0","0","0","0","0","0","0","0","0","0","0","0","1","0","0","0","0","0
","0","0","0","0","0","0","0","0","0","0","0","0","0","0","0","0","0","0","0","0","0","0","0","0","0
","0","0","1","1","0","0","0","0","0","0","0","0","0","0","0","1","0","0","0","0","0","0","0","0","0
","0","0","0","0","0","0","0","0","0","0","1","0","0","0","0","0","0","14","5","0","NID","tested_p
ositive"

"52","154","80","20","74","1","1","1","0","1","0","0","0","1","1","1","1","1","1","1","1","0",
"1","1","0","0","0","1","1","0","0","1","1","0","0","0","0","0","1","1","0","0","0","1","0","1","0",
"0","0","0","1","1","1","0","0","0","1","0","0","0","0","0","0","0","0","0","0","0","1","0","0","0",
"1","1","0","0","1","1","0","0","0","0","1","0","0","0","0","0","0","28","3","0","NID","tested_po
sitive"

"54","48","60","28","76","0","0","0","0","0","0","0","1","1","0","0","0","1","0","0","0","1","0","
0","0","0","0","0","0","0","0","0","0","1","0","0","0","0","0","0","0","0","0","0","0","1","0","0","
0","0","0","0","0","0","0","0","0","0","0","0","0","0","0","0","0","0","0","0","0","1","0","0","0","
0","0","0","0","0","0","0","0","0","0","1","0","0","0","0","0","0","14","3","0","NID","tested_neg
ative"

"48","77","70","26.5","79","0","0","0","0","1","0","0","0","0","0","0","0","1","1","0","0","0","0"
,"0","0","0","0","0","0","0","0","0","0","0","0","0","0","0","0","0","0","0","0","0","0","0","0","0"
,"0","0","0","0","0","0","0","0","0","0","0","0","0","0","0","0","0","0","0","0","0","0","0","0","0"
,"0","0","0","0","0","0","0","0","0","0","0","0","0","0","0","0","0","42","2","0","NID","tested_ne
gative"

"58","80","50","20","64","0","1","0","0","0","0","0","1","0","0","0","1","0","1","1","0","0","0","
1","0","0","0","0","0","0","0","0","1","1","0","0","1","0","0","0","0","0","0","0","0","0","1","0","
0","0","0","0","0","0","0","0","0","0","0","0","0","0","0","0","0","0","0","0","0","0","0","0","0","
0","0","0","0","0","0","0","0","0","1","0","0","0","0","0","0","0","7","1","0","NID","tested_nega
tive"

"62","122","80","19.5","77","0","1","1","0","1","0","0","0","0","0","0","0","1","1","0","1","0","0
","0","0","0","0","1","1","1","0","0","1","1","1","0","0","0","0","1","1","0","0","0","0","1","1","0
","0","0","0","0","0","1","0","0","0","1","1","0","1","0","0","0","0","0","0","0","1","0","0","0
","0","0","1","1","1","1","1","0","0","1","0","0","0","0","0","0","0","7","12","0","NID","tested_p
ositive"

"31","99","60","23","74","0","1","1","1","1","0","0","1","0","0","1","0","1","1","0","0","1","1","
0","0","1","0","0","1","1","0","0","1","1","1","0","0","0","0","1","0","1","0","0","0","1","1","0","
1","0","0","0","0","0","1","0","0","1","1","0","0","0","0","0","0","0","0","0","0","1","0","0","0","
1","1","0","1","1","1","1","0","0","1","1","0","0","0","0","0","0","49","4","0","NID","tested_neg
ative"

"43","210","70","16.5","49","0","0","1","0","1","1","0","0","0","1","0","0","1","0","0","0","0","0
","0","0","0","0","0","0","0","0","0","0","0","0","0","0","0","0","0","0","0","0","0","0","0","0","0
","0","0","0","0","0","0","0","0","0","0","1","0","0","0","0","0","0","0","0","0","0","0","0","0","0
","0","0","0","0","0","0","0","0","0","0","0","0","0","0","0","0","0","56","2","0","NID","tested_p

ositive"

"76","54","90","14","34","1","1","1","1","1","1","1","0","0","0","0","0","0","1","1","1","0","0","
0","0","0","0","0","0","0","0","0","1","1","0","0","0","0","0","1","1","0","1","1","0","1","1","0","
0","0","0","1","0","0","0","0","1","0","1","0","0","0","0","0","1","0","0","0","0","1","0","0","0","
0","0","0","0","1","0","0","0","0","0","0","0","0","0","0","0","0","60","1","0","NID","tested_neg
ative"

"44","41","70","19.5","56","1","1","0","0","0","0","0","0","0","0","0","0","0","1","0","0","0","0"
,"0","0","0","0","0","0","0","0","0","1","1","0","0","0","0","0","0","1","1","1","0","0","1","1","0"
,"0","0","0","0","0","0","0","1","1","1","0","0","0","0","0","0","0","0","1","0","0","1","0","0","0"
,"0","0","0","0","1","0","0","0","0","0","0","0","0","0","0","0","0","90","2","2","NID","tested_ne
gative"

"23","188","60","16","47","1","1","0","0","0","0","0","0","0","0","1","0","1","1","0","0","0","0",
"0","0","0","0","0","0","0","0","0","1","1","1","0","0","0","0","0","0","0","0","0","1","1","0",
"0","0","0","0","0","1","0","0","0","1","0","0","0","1","0","0","1","0","0","0","0","0","0","0","0",
"0","0","0","0","0","0","0","0","0","1","0","0","0","0","0","0","0","14","6","0","NID","tested_po
sitive"

"41","190","70","19","61","1","1","0","1","1","0","1","0","0","0","0","0","0","1","1","1","0","0",
"0","0","0","0","0","0","0","0","0","1","1","1","0","0","0","0","0","1","0","1","0","0","1","1","0",
"0","0","0","0","0","1","0","0","0","1","0","0","1","0","0","0","0","0","0","0","0","1","0","0","0",
"0","0","0","0","0","0","0","0","0","1","0","1","0","0","0","0","1","7","6","0","NID","tested_posi
tive"

"38","117","80","27","88","1","1","1","0","0","0","0","0","0","0","1","0","0","1","0","0","0","0",
"0","0","0","0","0","0","0","0","0","1","1","0","0","0","0","0","0","0","0","0","0","0","1","0","0",
"0","0","0","0","0","0","0","0","0","0","0","0","0","0","0","0","0","0","1","0","0","0","0","0","0",
"0","0","0","0","0","1","0","0","0","0","0","0","0","0","0","0","0","80","6","6","NID","tested_po
sitive"

"55","126","80","24","74","1","1","0","0","0","0","0","0","0","0","0","0","0","0","1","1","1","1",
"0","0","0","1","0","0","0","0","0","1","1","0","0","0","0","0","0","1","0","0","0","0","0","0","0",
"0","0","0","0","0","1","0","0","0","0","0","0","0","0","0","0","0","0","0","0","0","0","0","0","0",
"0","0","0","0","0","0","0","0","0","0","0","0","0","0","0","0","0","30","3","0","NID","tested_po
sitive"

"65","119","70","21","68","0","1","0","0","1","0","0","0","0","0","1","0","0","0","1","0","0","0",
"0","0","0","0","0","0","0","0","0","1","0","0","0","0","0","0","0","0","0","0","0","0","0","0","0",
"0","0","0","0","0","0","0","0","0","0","0","0","1","0","0","0","0","0","0","0","0","0","0","0","0",

"0","0","0","0","0","0","0","0","0","0","0","0","0","0","0","0","0","28","2","0","NID","tested_positive"

"48","39","60","33","95","1","1","0","1","1","0","0","0","0","0","1","0","0","0","0","0","0","0","0","0","0","0","0","0","0","0","0","1","0","0","0","0","0","0","0","0","0","0","0","0","0","0","0","0","0","0","0","0","0","0","0","0","0","0","0","0","0","0","0","0","0","0","0","0","0","0","0","0","0","0","0","0","0","0","0","0","0","0","0","0","0","0","0","0","0","0","0","0","1","0","0","0","0","1","14","5","3","GTD","tested_negative"

"36","173","80","25","89","1","1","1","1","0","0","0","0","0","0","1","1","1","1","1","1","1","1","1","1","1","1","1","1","1","1","0","1","1","0","1","0","0","0","0","1","1","1","0","0","1","1","1","1","0","0","1","0","1","1","1","0","1","1","1","1","0","0","1","0","0","1","0","0","0","0","0","0","0","0","0","0","1","0","0","0","0","1","0","0","0","0","0","0","0","30","6","1","NID","tested_positive"

"57","90","70","31","92","1","1","1","0","0","0","0","0","0","0","0","0","0","0","0","0","0","0","0","0","0","0","0","0","0","0","0","1","0","0","0","0","0","0","0","0","0","0","0","1","0","0","0","0","0","0","0","0","0","0","0","0","0","0","0","0","0","0","0","0","0","0","0","0","0","0","0","0","0","0","0","0","0","0","0","0","0","0","0","0","0","0","0","0","0","0","0","0","0","0","0","0","7","6","2","NID","tested_negative"

"34","155","90","19","59","1","0","0","1","0","0","0","0","0","0","0","0","1","0","0","0","1","0","0","0","0","0","0","0","0","0","0","1","0","0","0","0","1","0","1","0","1","0","0","0","0","0","0","0","0","1","0","1","0","0","0","1","0","0","0","0","0","0","0","0","0","0","0","1","0","0","0","0","0","0","0","1","0","0","0","0","1","0","1","0","0","0","0","1","14","2","0","NID","tested_positive"

"56","64","60","30","87","1","1","1","0","0","0","0","0","0","0","0","0","0","1","0","0","0","0","0","0","0","0","0","0","0","0","0","1","1","0","0","0","0","0","0","0","0","0","0","0","1","0","0","0","0","0","0","0","0","0","0","0","0","0","0","0","0","0","0","0","0","0","0","0","0","0","0","0","0","0","0","0","0","0","0","0","0","0","0","0","1","0","0","0","0","0","0","0","60","3","1","NID","tested_negative"

"28","211","86","19","57","1","1","1","1","0","1","0","0","1","0","1","1","1","1","1","0","1","1","1","0","1","0","1","1","0","1","0","1","1","1","1","0","0","1","1","1","1","0","0","1","1","1","1","0","1","0","0","1","1","1","0","0","1","0","1","1","1","1","1","0","1","1","0","0","0","0","1","0","1","0","0","1","1","1","0","1","0","1","1","0","0","0","0","0","0","35","3","2","NID","tested_positive"

"48","161","90","17","50","1","1","1","1","1","1","1","1","0","0","0","0","0","1","1","0","0","1","1","1","0","0","0","0","0","1","0","1","1","0","0","1","0","0","1","0","0","1","1","1","1","1","0",

"0","1","0","1","0","0","0","0","0","1","0","0","1","0","0","0","0","0","0","0","0","0","0","0",
"1","1","0","0","1","1","0","0","0","1","0","0","0","0","0","0","0","37","5","3","GTD","tested_po
sitive"

"32","131","60","28","81","1","1","1","1","0","1","0","0","1","0","1","0","0","1","1","1","0","1",
"0","0","0","1","0","1","1","1","0","1","1","1","0","0","0","0","0","0","0","1","0","1","1","0","1",
"1","1","0","0","0","0","0","0","1","1","0","1","1","0","0","0","0","0","0","0","1","0","1","1",
"0","0","0","0","1","1","0","0","1","1","0","0","0","0","0","0","1","21","8","4","GTD","tested_po
sitive"

"30","142","80","16.5","43","0","0","0","0","1","1","1","0","0","0","1","0","0","0","0","0","0","1
","1","0","0","0","0","0","0","0","0","0","0","1","1","1","0","0","0","0","0","1","0","0","0","0","0
","0","0","0","1","0","0","0","0","0","0","0","0","1","0","0","0","0","0","1","0","1","0","0","0","0
","0","0","0","0","0","0","0","1","1","0","0","0","1","0","0","1","0","30","3","2","IND","tested_p
ositive"

"55","39","70","23","67","0","1","1","0","0","1","0","0","1","0","0","1","0","1","0","1","0","0","
1","0","0","1","0","0","0","0","0","1","0","0","0","0","0","0","0","1","0","0","0","1","0","0","0","
0","0","0","0","0","1","0","0","1","0","0","0","1","0","0","1","0","0","0","0","0","0","0","0","0","
1","0","0","0","0","1","0","0","1","0","0","0","1","0","0","0","0","30","2","0","NID","tested_neg
ative"

"54","171","90","19","69","1","1","0","1","0","1","0","1","0","1","1","0","0","0","1","0","1","0",
"1","0","0","0","0","1","1","0","1","1","1","0","0","1","0","0","0","0","1","0","1","0","0","0","1",
"0","0","0","1","1","0","1","0","1","0","0","1","0","0","0","0","0","0","0","0","1","1","0","0","0",
"0","1","0","1","1","0","0","1","0","1","0","0","0","0","0","0","0","28","3","1","NID","tested_po
sitive"

"47","128","60","21","63","1","1","1","1","0","1","0","0","0","0","0","0","1","1","1","1","1","0",
"0","0","1","1","0","1","1","1","0","1","1","0","0","1","1","1","0","1","0","0","1","1","1","1","0",
"0","0","0","0","0","0","0","1","1","1","0","0","1","0","0","0","1","1","1","1","1","1","0","0",
"0","0","0","0","1","1","1","0","0","1","1","1","0","0","0","0","0","28","4","1","NID","tested_po
sitive"

"67","118","94","19.5","61","0","1","1","0","0","1","0","0","0","1","1","0","1","0","1","0","1","0
","1","1","1","0","0","0","0","0","0","1","0","0","1","0","0","0","1","0","0","0","0","1","0","1","0
","0","0","0","1","1","0","0","0","0","1","0","0","0","0","1","0","0","0","0","0","1","0","1","0","0
","0","1","0","0","1","1","0","0","0","1","1","0","0","0","0","0","0","90","2","0","NID","tested_p
ositive"

"56","161","80","19","62","1","1","1","1","1","1","0","0","0","0","1","1","1","1","1","1","1","1",

"0","1","0","1","0","0","1","0","0","1","1","0","0","0","0","0","1","1","0","1","0","1","1","1","0",
"0","0","0","1","1","1","0","0","0","1","1","0","0","0","0","1","0","0","1","0","0","1","1","1","1",
"0","0","0","0","1","1","0","0","0","1","0","0","0","1","1","0","0","28","2","0","NID","tested_po
sitive"
